# Braving the waves: exploring capability well-being patterns in seven European countries during the COVID-19 pandemic

**DOI:** 10.1007/s10198-023-01604-8

**Published:** 2023-07-06

**Authors:** Sebastian Himmler, Job van Exel, Werner Brouwer, Sebastian Neumann-Böhme, Iryna Sabat, Jonas Schreyögg, Tom Stargardt, Pedro Pita Barros, Aleksandra Torbica

**Affiliations:** 1 Erasmus School of Health Policy and Management, Erasmus University Rotterdam, P.O. Box 1738, 3000 DR Rotterdam, The Netherlands; 2 Erasmus Centre for Health Economics Research (EsCHER), Erasmus University Rotterdam, Rotterdam, The Netherlands; 3 Hamburg Center for Health Economics, University of Hamburg, Esplanade 36, 20354 Hamburg, Germany; 4 Nova School of Business and Economics, R. Holanda 1, 2775-405 Carcavelos, Portugal; 5 Centre for Research On Health and Social Care Management, CERGAS, Bocconi University, Via Röntgen N. 1, 20136 Milan, Italy

**Keywords:** Well-being, Capability well-being, ICECAP-A, COVID-19, Pandemic, I31

## Abstract

The COVID-19 pandemic considerably impacted the lives of European citizens. This study aims to provide a nuanced picture of well-being patterns during the pandemic across Europe with a special focus on relevant socio-economic sub-groups. This observational study uses data from a repeated, cross-sectional, representative population survey with nine waves of data from seven European countries from April 2020 to January 2022. The analysis sample contains a total of 25,062 individuals providing 64,303 observations. Well-being is measured using the ICECAP-A, a multi-dimensional instrument for approximating capability well-being. Average levels of ICECAP-A index values and sub-dimension scores were calculated across waves, countries, and relevant sub-groups. In a fixed effects regression framework, associations of capability well-being with COVID-19 incidence, mortality, and the stringency of the imposed lockdown measures were estimated. Denmark, the Netherlands, and France experienced a U-shaped pattern in well-being (lowest point in winter 2020/21), while well-being in the UK, Germany, Portugal, and Italy followed an M-shape, with increases after April 2020, a drop in winter 2020, a recovery in the summer of 2021, and a decline in winter 2021. However, observed average well-being reductions were generally small. The largest declines were found in the well-being dimensions attachment and enjoyment and among individuals with a younger age, a financially unstable situation, and lower health. COVID-19 mortality was consistently negatively associated with capability well-being and its sub-dimensions, while stringency and incidence rate were generally not significantly associated with well-being. Further investigation is needed to understand underlying mechanisms of presented patterns.

**Supplementary Information:**

The online version contains supplementary material available at 10.1007/s10198-023-01604-8.

## Introduction

The COVID-19 pandemic challenged health systems and societies alike. As the impact of COVID-19 on the physical health of populations in Europe became clearer [1], it was increasingly mitigated through the vaccination programs starting in December 2020 and other government measures. To date, less is known about the consequences of the pandemic and related government measures [2] on the well-being of populations in Europe.

### Overview of findings on well-being changes

Previous research relating to the well-being impacts of the pandemic mainly focused on mental health and life satisfaction. International reviews that summarized studies from 2020, the first year of the pandemic, found that the pandemic had caused an increase in mental health issues. Psychological distress was more frequently observed and scores of anxiety and depression were higher compared to before COVID-19 [3]. One multi-country study found high prevalence rates for probable anxiety and probable depression in Europe throughout the pandemic, with younger age groups being at higher risk [4]. Another cross-country study confirmed this pattern for the early phase of the pandemic, while also showing evidence for problems with self-confidence and social connectedness [5]. It is worth mentioning here also the direct negative mental health impact of getting infected with COVID-19 [6]. Another more recent international review also confirmed negative mental health changes, while suicide rates and life satisfaction appeared to be constant [7]. A further review summarized the negative psychological impact of COVID-19 lockdowns as small in magnitude and highly heterogeneous across sub-groups [8]. Comparing pre-pandemic statistics from the World Happiness Report to values from 2020 revealed higher frequencies of negative emotions but fairly stable average life satisfaction, especially in Europe [9].

### Well-being findings for specific sub-groups

Several characteristics associated with a more negative mental health or well-being impact of the pandemic were identified in the literature. Cross-sectional and longitudinal evidence from the UK points toward more negative well-being patterns in younger age groups, women, people with poor mental or physical health, and people with lower socio-economic status [10–13]. Cross-sectional data from Spain confirmed most of these findings [14]. A large-scale study from the US found that the impact of lockdowns on mental health was especially pronounced in women [15]*. International studies found high rates of mental issues among students, again concentrated among females* [16, 17]. Smaller impacts in older age groups compared to younger age groups were found in Australia and the Netherlands [18, 19]. A multi-country study from Europe highlighted that financial instability was related to lower well-being during the first months of pandemic restrictions [20]. This was also observed in a study from Spain [14]. It was also shown that the relevance of financial factors for individuals’ happiness increased during the pandemic [21].

### Research gap and objective

These studies have in common that they mostly examined the initial phase of the pandemic, were limited to a specific country or population group, addressed only overall well-being [22], and consisted of either a single cross-section or spanned a limited number of data waves. This study has two aims: first, to contribute to the existing literature by examining the development of overall well-being, more specifically capability well-being, and separate dimensions thereof, over the whole course of the COVID-19 pandemic in multiple European countries using a unique dataset. Second, this study examines to what extent capability well-being and its dimensions were associated with COVID-19 incidence, mortality, and the stringency of the imposed lockdown measures. The contribution, therefore, lies in providing a, to date, unparalleled, detailed overview of capability well-being patterns, across well-being dimensions, countries, and sub-groups, and a first exploration of correlating factors of these patterns over the first 2 years of the COVID-19 pandemic.

## Methods

### Setting and participants

To examine how well-being developed across Europe during the pandemic, we used data from the European COvid Survey, or ‘ECOS’ [23]. This is a high-frequency, repeated cross-sectional survey, which investigates COVID-19-related topics across seven central and western European countries about every 3 months (United Kingdom, Portugal, the Netherlands, Italy, France, Germany, and Denmark). The main body of the survey questions remained constant across all waves. The first nine waves of ECOS were collected in April, June, September, and November 2020, and January, April, June, September, and December 2021/January 2022. The survey was administered online. Participants were recruited using multi-sourced online panels provided by the market research company Dynata. Each of the nine waves of the survey included 1000 respondents from each of the seven included countries. The country samples were quota-sampled to be representative for the adult population of each country in terms of region, age categories, gender, and education. To ensure the comparability of the questionnaire across countries, the survey was translated from English into the six respective languages by native speakers. Informed consent was obtained from all survey respondents. The ECOS research project and the corresponding data collection received ethical approval from the *blinded*.

The number of observations across the nine waves and the seven countries included in all waves was 64,303. Of these, 51,094 stem from 11,857 individuals, who participated in multiple waves, while 13,209 observations were from individuals who participated once, providing a partially longitudinal character.

**Fig. 1:**
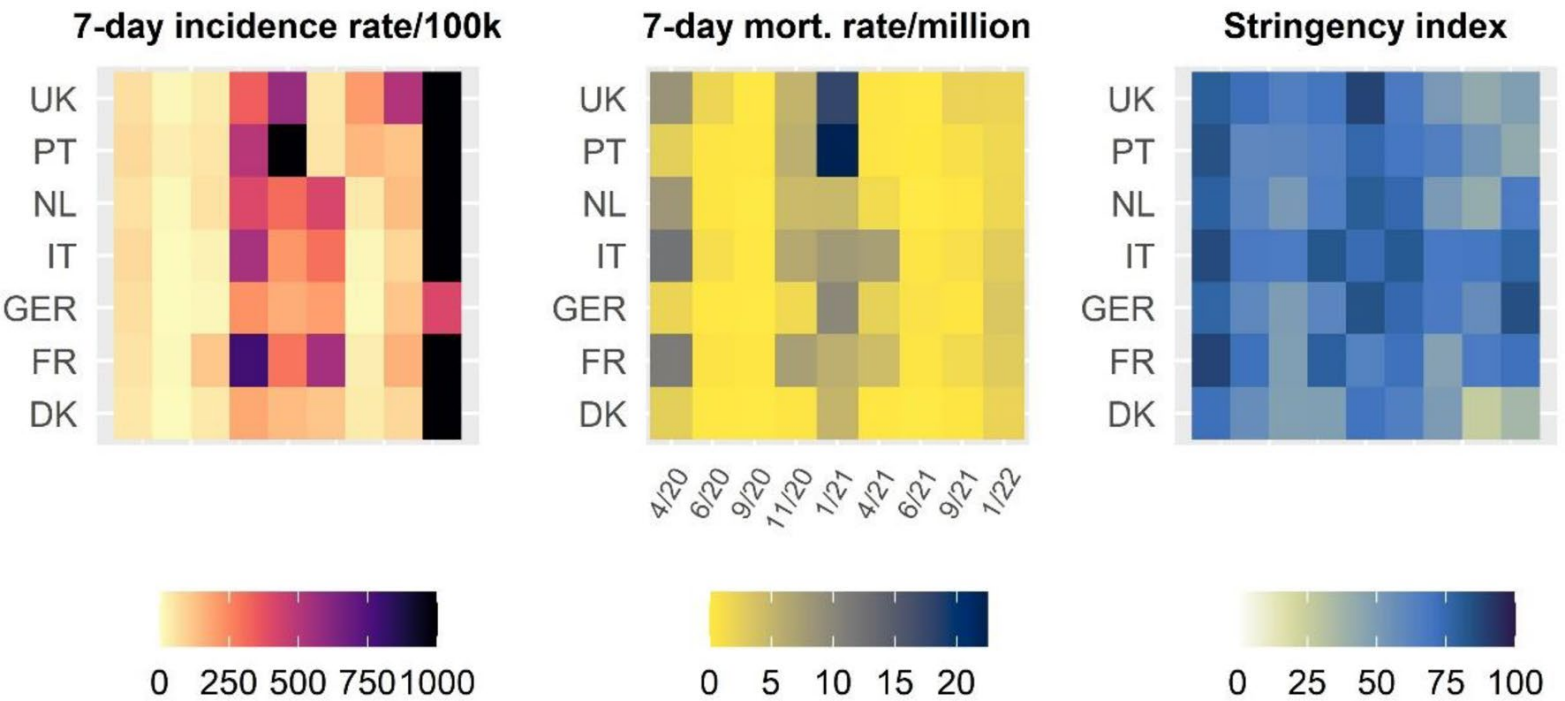
Mean incidence rate, mortality rate, and 14-day stringency index across the seven countries and the nine waves of data collected from April 2020 to September 2021. Incidence rate capped at 1000 (black)

To provide some context to the nine waves of the ECOS survey, Fig. 1 provides information about the severity of the pandemic in the seven countries in terms of the means of 7-day incidence and 7-day mortality per 1 million inhabitants extracted from Our World in Data [24], and the 14-day stringency of government measures to contain the pandemic based on the Oxford COVID-19 Government Response Tracker [2]. Officially reported incidence rates were comparatively low during the first three waves (April to September 2020), while mortality rates were at a high level, especially in France and Italy. Mortality rates peaked during waves four to six (November 2020–April 2021), with 7- day mortality rates per 1 million population increasing up to 17.7 and 22.2 in the UK and Portugal, respectively. The stringency and duration of government measures were also highest during this period, while measures were relaxed over the last three waves of data collection in most countries (with Germany as an exception).

### Well-being measure

The well-being measure used in this study is the ICEpop CAPability measure for Adults (ICECAP-A) [25]. Capability well-being is a class of subjective measures concerned with an individual’s ability to live the sort of life he or she is interested in, relating to the work by Amartya Sen [26]. The ICECAP-A’s five components are specifically selected to reflect capability well-being among adults. Therefore, this measure may be particularly useful for our purposes, as the pandemic and the government responses are restricting people’s capability to live the sort of life that interests them. The five distinct dimensions of capability well-being captured by the ICECAP-A are the following: stability (an ability to feel settled and secure), attachment (an ability to have love, friendship and support), autonomy (an ability to be independent), achievement (an ability to achieve and progress in life), and enjoyment (an ability to experience enjoyment and pleasure) [25]. Respondents are asked to indicate their capability level in each of these five dimension using a four-point Likert scale, scored from 1 (absence of capability) to 4 (full capability). The ICECAP-A has been validated in different contexts and countries [27–31], and is increasingly used in economic evaluations that take well-being as measure of outcome [31]. Prior to the first wave of ECOS, official translations of the ICECAP-A were available for all included countries, except for Portugal. The Portuguese version used in this study was created using back-and-forth translation conducted by Portuguese native speakers of the ECOS study group.

In the descriptive analysis, we used responses to the five ICECAP-A questions in two ways: first, a summary index for capability well-being ranging from 0 (no capability) to 1 (full capability) was calculated based on weights derived for the UK, as no such weights exist for Portugal, Italy, France, Germany, or Denmark [32], while weights for the Netherlands only became recently available [33]. Second, we used the four-point Likert scale for the separate dimensions ranging from 1 (best) to 4 (worst).

### Sub-groups

As our objective was to investigate well-being developments also across sub-groups of interest, we extracted information from the ECOS data on several individual characteristics selected based on the related literature summarized in the introduction: age, gender, education, financial security (being able to make ends meet), and health status. Age was categorized into three groups with comparable samples sizes (18–34, 35–64, 65 and above). Education was coded into three levels (low, middle, high), with ‘high’ education implying some form of university education (see Appendix 1 for an overview of the education classification system and categorization for all included countries). The caveat of such a classification is that educational attainment is inherently difficult to compare across countries.

If respondents indicated that their household had ‘some’ or ‘great’ difficulty in making ends meet (as opposed to ‘fairly easily’ and ‘easily’), their situation was categorized as ‘financially insecure’. Health status was assessed using official translations of the EQ-5D-5L [34]. An unadjusted sum score was calculated by summing the five-point Likert scale responses across the five dimensions, resulting in a health problems index score ranging from 5 (no problems) to 25 (extreme problems on all dimensions).

### Statistical analysis

The descriptive analysis of this rich set of capability well-being data consists of three steps. First, to provide an overview of the general trends in capability well-being across countries during the COVID-19 pandemic, mean ICECAP-A utility index scores and 95% confidence intervals were calculated for each wave and country, and plotted alongside the mean COVID-19 mortality rate. Second, mean country and wave specific Likert scale scores of the five ICECAP-A dimensions were computed and mapped to illustrate differences in trends for the five dimensions across countries and waves. Third, mean ICECAP-A utility index scores and 95% confidence intervals across countries and waves were calculated and plotted for sub-groups defined by age, gender, education, financial security, and health status.

To explore correlations between COVID-19-related factors and the observed well-being patterns, regression analyses were conducted pooling observations across countries. This entailed regressing 7-day COVID-19 incidence rate per 100,000, 7-day COVID-19 mortality rate per million, and 14-day government stringency index on country level on ICECAP-A utility values. Linear fixed effects regressions were used, controlling for individual and wave fixed effects and clustering standard errors on the individual level. A similar approach has previously been applied to life satisfaction data [35]. In a second step, separate regressions were conducted including interactions between the three variables of interest and sub-groups defined by age, gender, education, financial security, and health status.

The abovementioned steps of the regression analyses were repeated for each of the five ICECAP-A dimensions as dependent variable, instead of the overall utility index. To make regression estimates comparable to the initial analysis, the dimension scores were linearly rescaled to the same 0 (no capability) to 1 (full capability) range. The regressions analysis was conducted in Stata 17 (Stata Corp.).

## Results

A total of 64,303 observations from 25,062 individuals in the ECOS data were used for the analysis. The only exclusion criteria applied was missing data in one of the five ICECAP-A dimensions (*n* = 164). Appendix Table A2 contains descriptive information for the country samples and the total sample. Next to expected differences in the sampling variables (age, gender, and education), financial insecurity varied considerably across countries. It was not possible to achieve representativeness in terms of education in all countries and waves.

### Overall capability well-being patterns

**Fig. 2:**
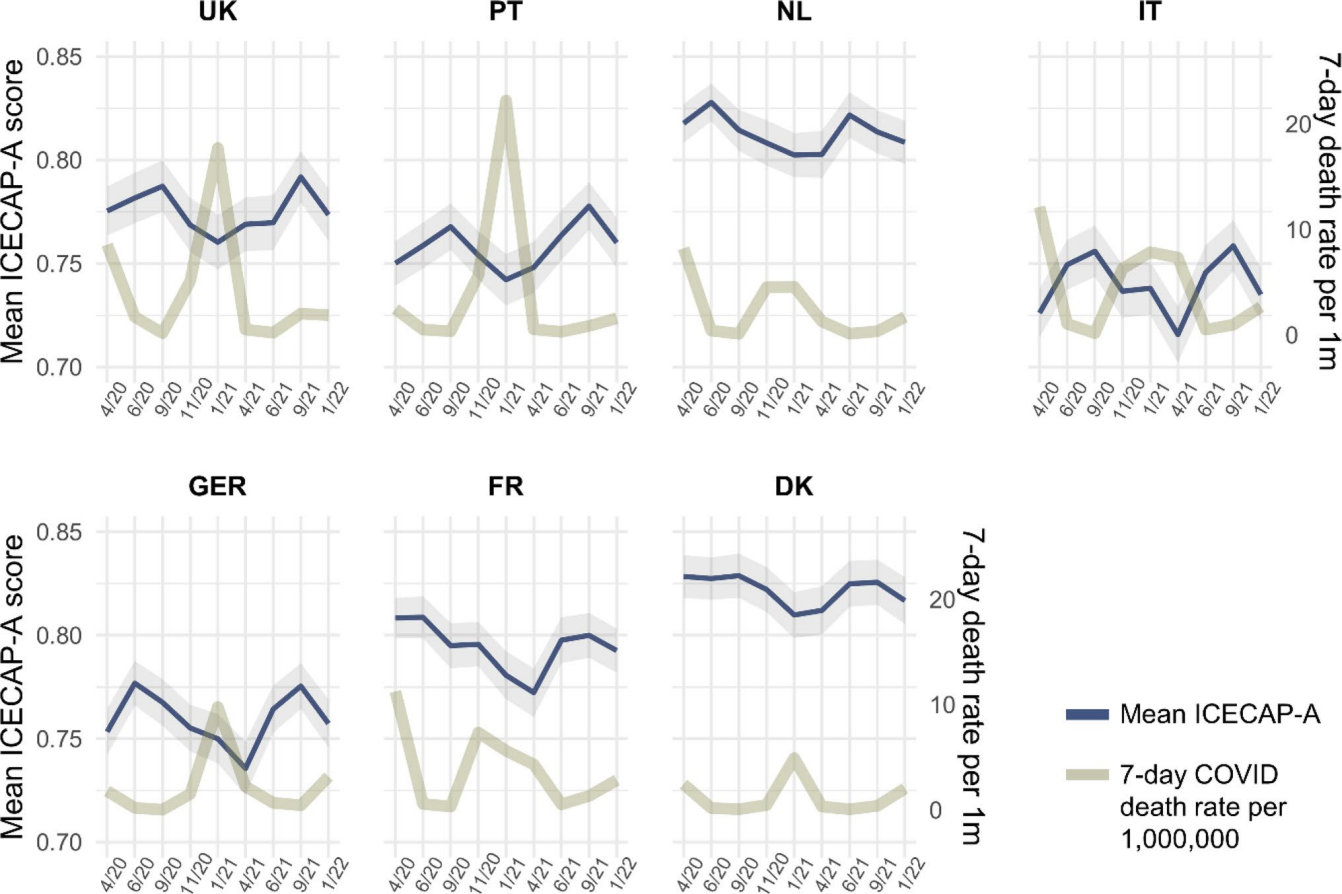
Mean ICECAP-A utility score across countries and waves of data collection. The utility score ranges from 0 (no capabilities) to 1 (full capabilities) and was calculated using weights derived for the UK [32]. Shaded areas represent 95% confidence intervals. Mortality data were extracted from Our World in Data [24]

The mean capability well-being scores across the nine waves of ECOS are plotted in Fig. 2. Mean ICECAP-A scores during the first wave of COVID-19 in April 2020 ranged from 0.73 (0.20) in Italy to 0.83 (0.17) in Denmark (see Appendix Table A3). Figure 2 shows that in countries with lower ICECAP-A scores in April 2020 (Italy, Portugal, Germany, and the UK), capability well-being recovered over the course of the summer of 2020. During the same period, capability well-being remained relatively constant in Denmark and the Netherlands. Across all countries, capability well-being decreased with the start of the autumn in 2020, coinciding with increases in COVID-19 mortality rates, and the implementation of government measures to contain the pandemic. The largest declines over the winter and early spring of 2021 were observed for Italy (from 0.76 to 0.72) and Germany (from 0.78 to 0.74). Average declines in capability well-being in Denmark and the Netherlands were modest. Well-being continued to decrease up until April 2021 in Italy, Germany and France, while it already increased again in the remaining countries. Afterward, well-being bounced back in all countries almost to September 2020 averages, with additional increases observed in September in Italy, Germany, Portugal, and the UK. In November, values again decreased, similar to the year before. Overall, well-being changes appeared to be inversely related to the severity of COVID-19, here illustrated using the COVID-19 mortality rate (Fig. 2).

### Absolute level of capability well-being across dimensions

**Fig. 3:**
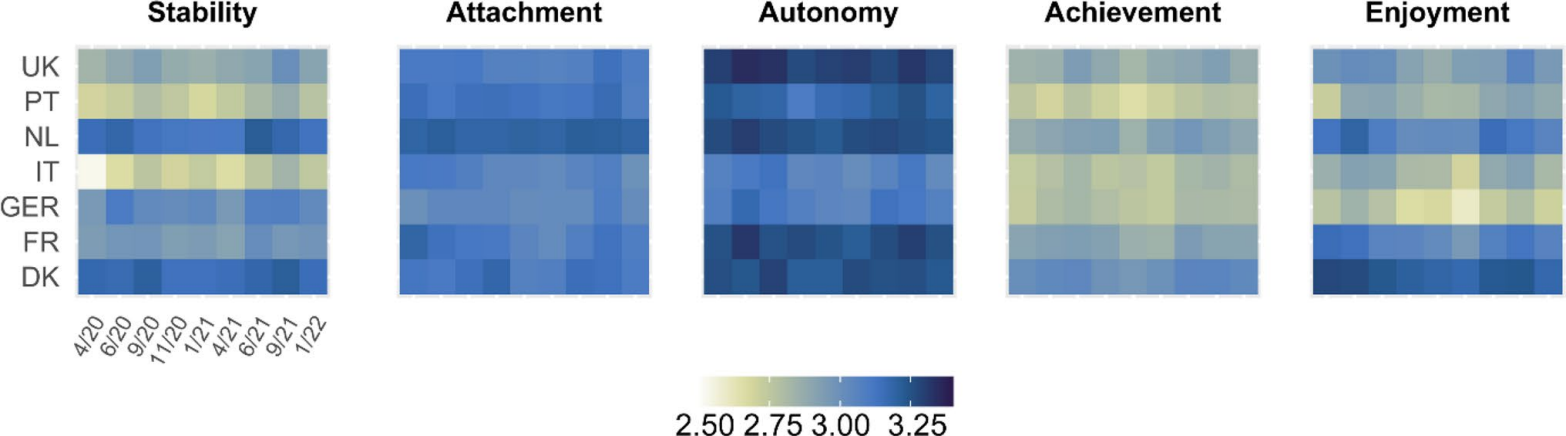
Mean ICECAP-A dimension scores across countries and waves of data collection. ICECAP-A dimension scores range from 1 (worst) to 4 (best). Number of observations per cell ~ 1000

A more detailed impression on the state of capability well-being across countries during the pandemic is provided in Fig. 3, which plots developments for the five well-being dimensions of the ICECAP-A separately. In April 2020, Italian (2.53, SD = 0.90) and Portuguese (2.70, SD = 0.80) samples scored lowest on the stability dimension, while the Dutch (3.16, SD = 0.70) and Danish samples (3.18, SD = 0.69) scored highest (see Appendix Table A3). Stability increased slightly over time in the Italian and Portuguese samples but remained lower compared to other countries. Respondents from the Netherlands and Denmark consistently scored highest in this dimension. Differences in scores between country samples and across time on the attachment dimension were less pronounced. Across waves, Dutch participants scored highest (3.20, SD = 0.77) and German participants scored lowest (3.04, SD = 0.82) on this dimension. The lowest scores on the autonomy dimension were consistently observed for the Italian (3.07, SD = 0.87) and German samples (3.10, SD = 0.78). The corresponding average across waves was highest in the UK sample (3.30, SD = 0.79). The samples from Germany (2.79, SD = 0.81), Italy (2.79, SD = 0.80), and Portugal (2.74, SD = 0.77) generally scored low on the achievement dimension, while the highest score was observed for the Danish respondents (3.02, SD = 0.76). The average score on the enjoyment dimension in the samples across waves was lowest in Germany (2.73, SD = 0.79) and Italy (2.85, SD = 0.81), and highest in Denmark (3.22, SD = 0.77). To highlight changes in the five dimensions of the ICECAP-A during the pandemic, Appendix Figure A1 plots the difference of means compared to the first wave of data collection in April 2020. Appendix Table A3 provides the reference values for April 2020 for both capability well-being and dimension scores across all countries.

### Changes in capability well-being across sub-groups

#### Age groups

**Fig. 4:**
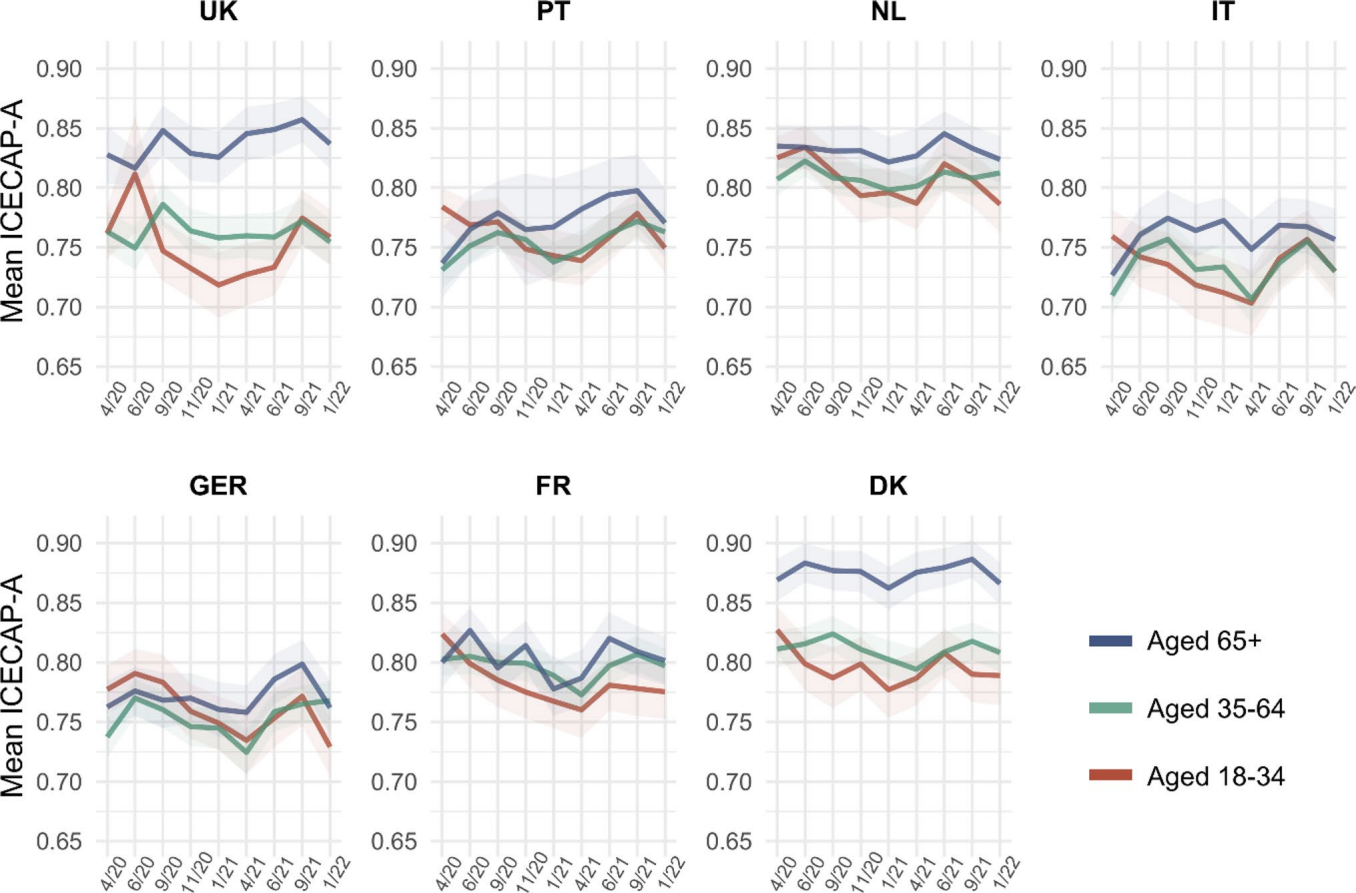
Mean ICECAP-A utility score across countries and waves of data collection, by age groups. The utility score ranges from 0 to 1 and was calculated using weights derived for the UK [32]. Shaded areas represent 95% confidence intervals

Well-being in the age group 65 + varied less as compared to other age groups across the pandemic (Fig. 4). Largest decreases could be observed in the age group 18–34 across all countries, in some instances reversing the ranking of well-being across age groups. Well-being differences between age groups were highest in the UK and Denmark.

When examining changes in mean ICECAP dimension scores compared to April 2020 differentiated by age groups (Appendix Figure A2), we found that dimension level changes moved largely in parallel between the older two age groups, which also observed the largest increases in the stability dimension. Furthermore, while generally all dimensions in the youngest age group were negatively affected, decreases in well-being were largest in the attachment and enjoyment dimension, especially in Italy and France.

#### Gender

**Fig. 5:**
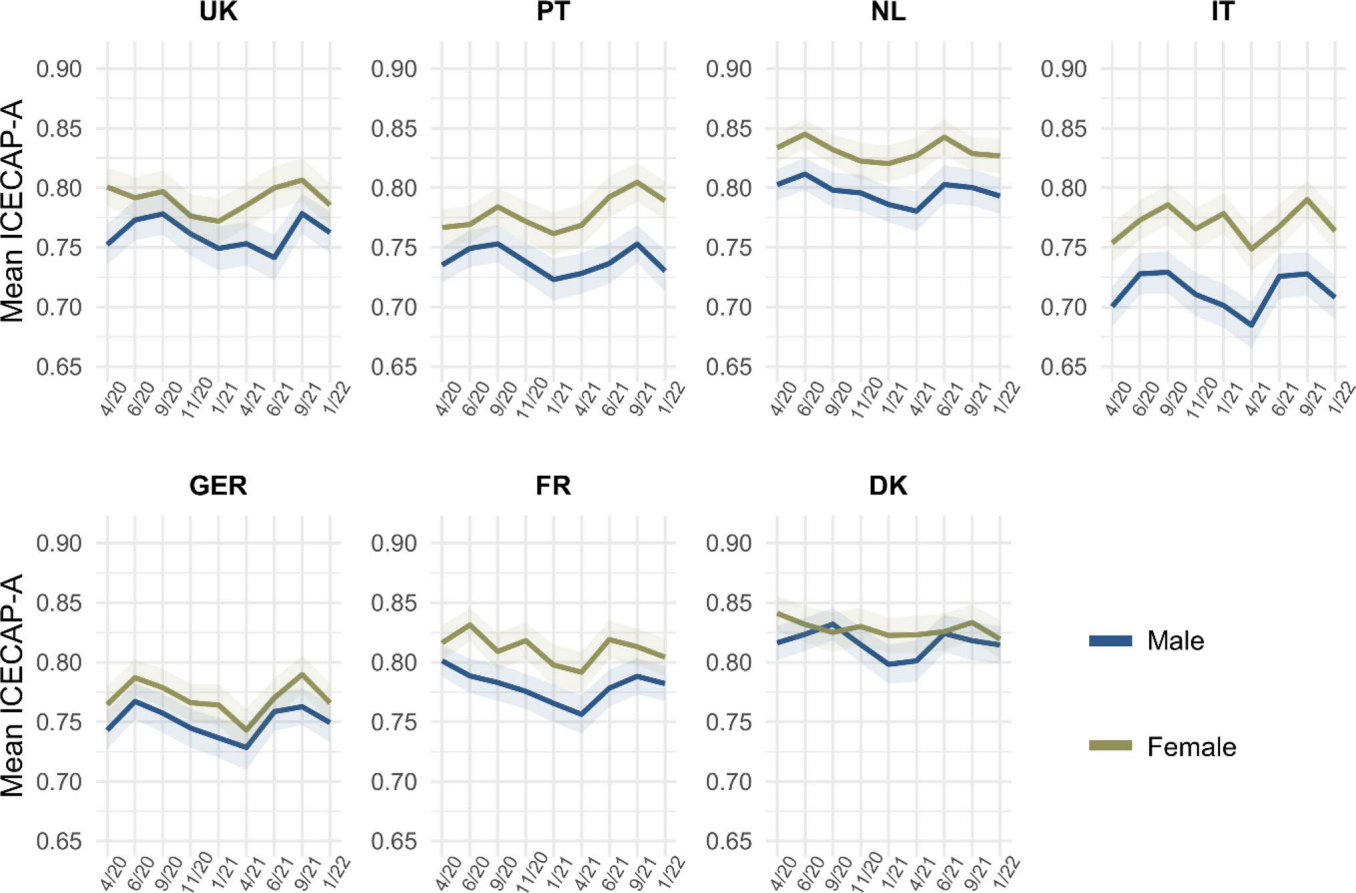
Mean ICECAP-A utility score across countries and waves of data collection, by gender. The utility score ranges from 0 to 1 and was calculated using weights derived for the UK [32]. Shaded areas represent 95% confidence intervals

Well-being was consistently higher among females, with the smallest difference observed in Denmark, and the largest difference in Italy and Portugal (Fig. 5). Changes in well-being for females and males generally followed the same pattern during the pandemic.

Changes in mean scores on the different dimension of the ICECAP-A as compared to the beginning of the pandemic did not differ considerably between females and males (Appendix Figure A3). Notable exceptions were that females in the UK, Portugal, and Germany experienced larger increases in the stability dimension as compared to April 2020, and larger decreases in the attachment dimension in Italy and in the autonomy dimension in Portugal. The least favorable trend for females across all dimensions (except for stability) could be observed in France.

#### Education levels

**Fig. 6:**
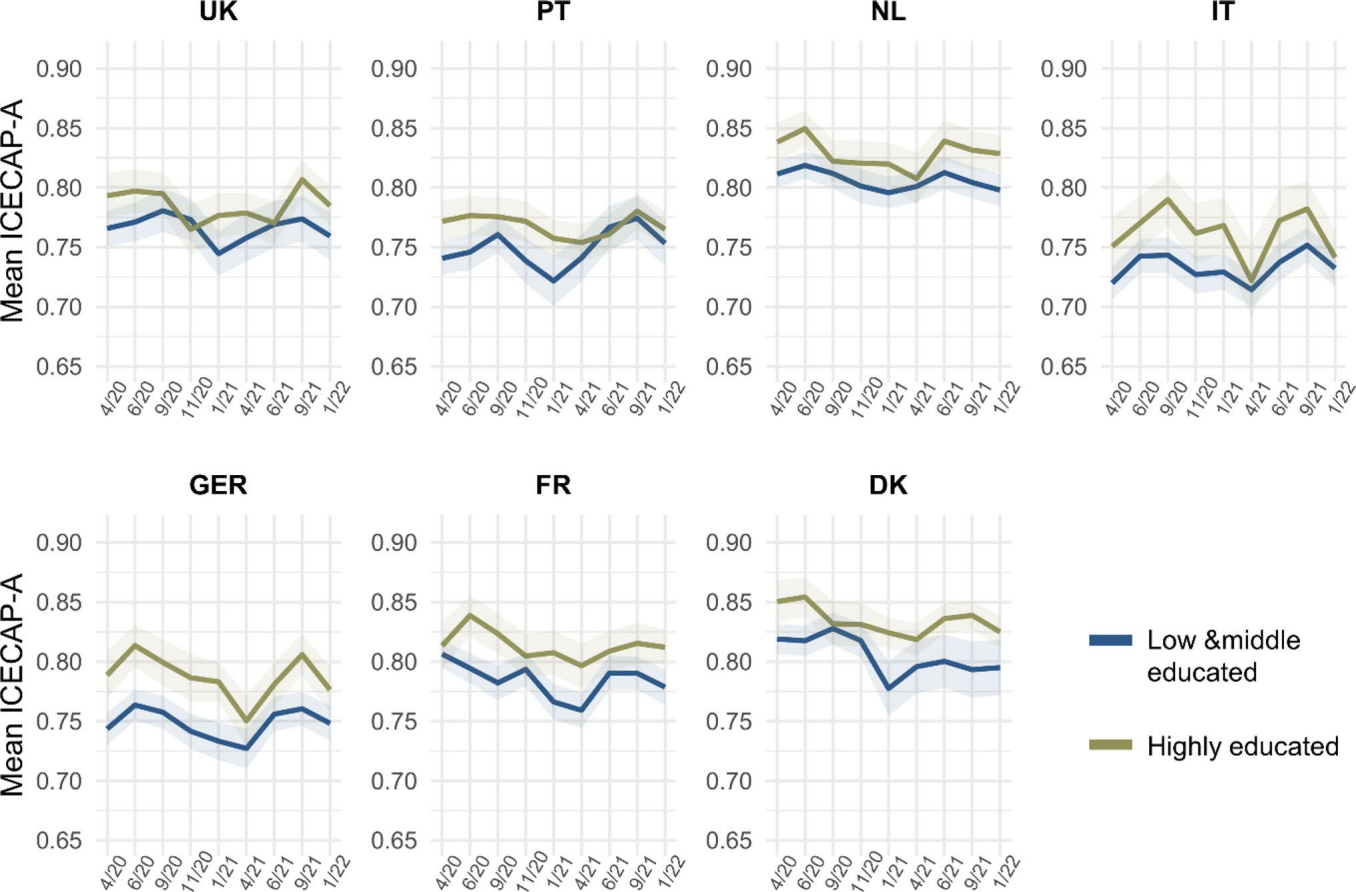
Mean ICECAP-A utility score across countries and waves of data collection, by education level. Highly educated corresponds to some form of tertiary education (see Appendix 1). The utility score ranges from 0 to 1 and was calculated using weights derived for the UK [32]. Shaded areas represent 95% confidence intervals

Low and middle level of formal education were combined due to low numbers of observations with low education in some countries (several instances of *n* < 100, especially in Portugal). Overall, capability well-being was generally higher for highly educated individuals (Fig. 6). Steeper drops in capability well-being were observed for the group of low and middle educated individuals in Portugal, France, and Denmark, while a somewhat reversed development could be observed for the Netherlands and Italy.

Regarding changes in the ICECAP-A dimensions, no overall diverging trends could be identified (Appendix Figure A4). The most pronounced gradient for education could be observed for France, where highly educated individuals fared better during the pandemic regarding the stability, attachment, autonomy, and achievement dimensions. Higher autonomy for highly educated individuals was also found for Germany. Interestingly, low and middle educated individuals in Portugal had a more positive pattern regarding stability and autonomy as compared to the highly educated. A similar trend, but less pronounced, could be observed in the UK for the attachment and autonomy dimensions.

#### Health status

**Fig. 7:**
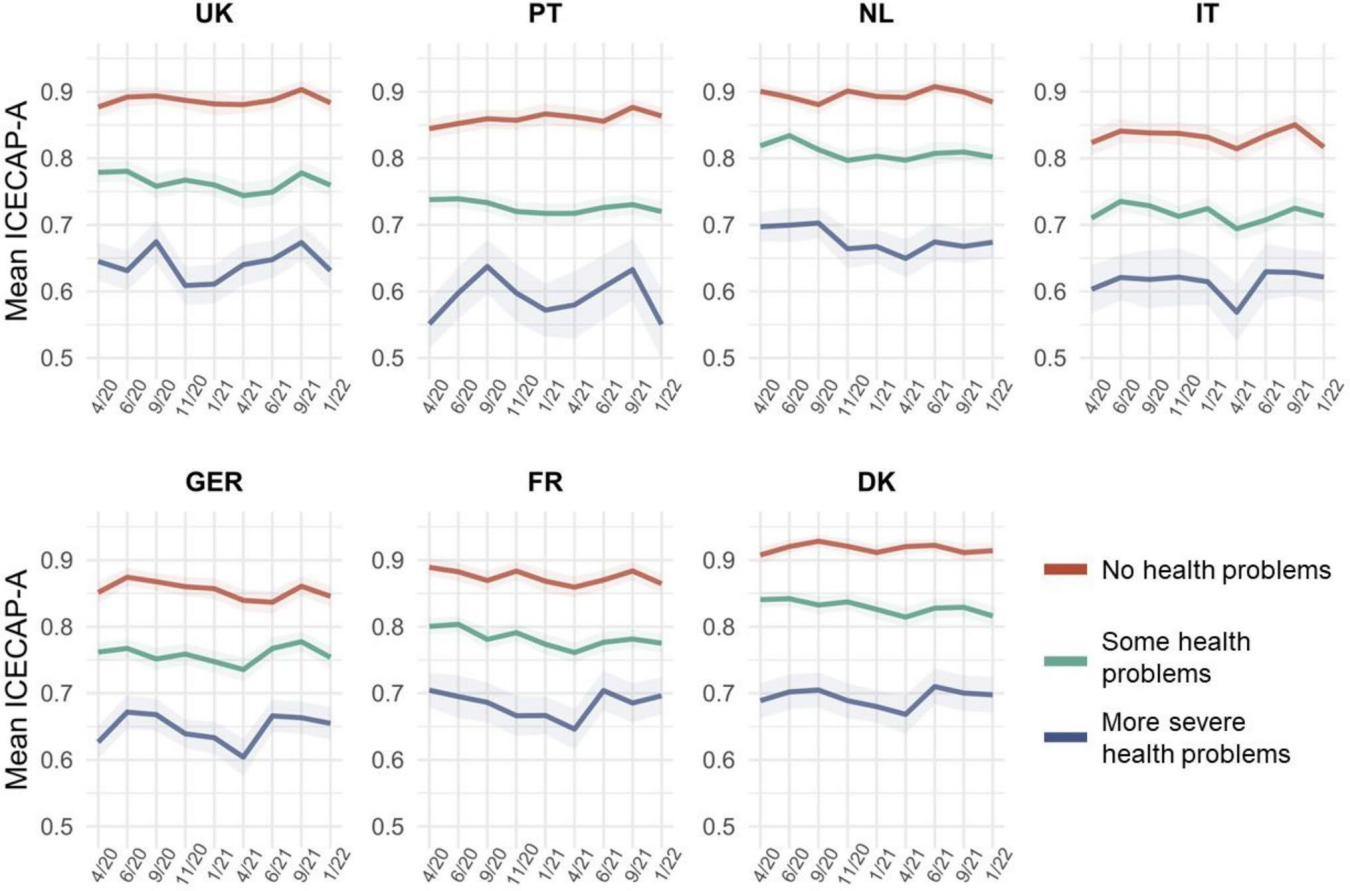
Mean ICECAP-A utility score across countries and waves of data collection, by health status using EQ-5D sum score. No health problems (5), some health problems (6–9), more severe health problems (10 +) on a theoretical range of 5 (no problems) to 25 (extreme problems on all dimensions). The utility score ranges from 0 to 1 and was calculated using weights derived for the UK [32]. Shaded areas represent 95% confidence intervals

Overall, capability well-being scores varied considerably according to health status (Fig. 7). The capability well-being of individuals with more severe health problems generally fluctuated more over the course of the pandemic in all countries as compared to individuals with no health problems, who experienced rather stable capability well-being. Most extreme fluctuation in the lower health group was observed for Portugal.

Changes in mean ICECAP-A dimension scores as compared to April 2020, differentiated by the health status of individuals, are plotted in Appendix Figure A5. Overall, the largest positive and negative changes occurred in the least healthy group, which for instance experienced larger increases in stability (except for the Netherlands). In the UK, individuals with lower health status suffered more in terms of attachment as compared to individuals with higher health status. Similar differential effects could also be observed for autonomy in Germany and France, and for achievement in the Netherlands. Conversely, negative changes in enjoyment were less frequently observed in the least healthy group.

#### Financial security

**Fig. 8:**
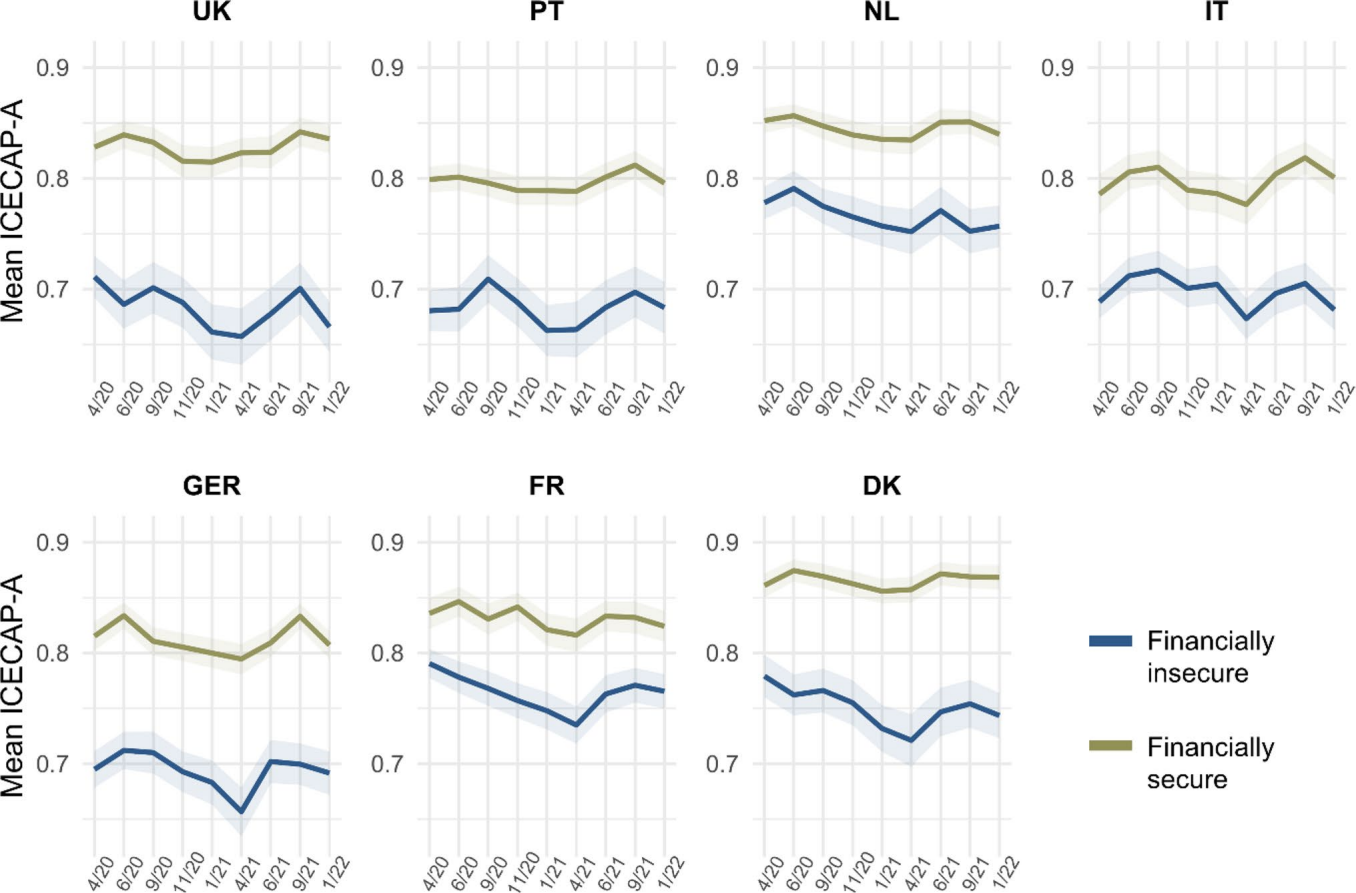
Mean ICECAP-A utility score across countries and waves of data collection, by financial security. “Insecure” defined as having some or great difficulties with making ends meet. The utility score ranges from 0 to 1 and was calculated using weights derived for the UK [32]. Shaded areas represent 95% confidence intervals

Individuals who had no difficulties in making ends meet consistently had higher capability well-being in all countries, with the largest gap in the UK and the smallest gap in the Netherlands and France (Fig. 8). Overall, the capability well-being of individuals with lower financial security was more heavily impacted in most countries, with the gap widening especially in the UK and Denmark.

Plotting changes in mean ICECAP-A dimension scores as compared to April 2020 differentiated by financial security (Appendix Figure A6), one first general observation is that declines in attachment, autonomy, and achievement were mostly observed in individuals with lower financial security. At the same time, individuals with higher financial security overall experienced only smaller reductions or even increases in these well-being dimensions during the pandemic. This trend was strongest in the UK, Portugal, Denmark, and France, while it could not be observed for Germany (and, to a lesser extent, the Netherlands). Reductions in enjoyment were observed across the two sub-groups, while generally more pronounced among individuals with lower financial security. The stability dimension was more negatively affected in the same sub-group in France and Denmark.

### Results from the regression analysis

Pooling all observations across countries and controlling for individual and wave fixed effect, we found that stringency index and incidence rate were not significantly associated with ICECAP-A utility scores (Table 1, column 1), while an increase in mortality rate by 10 per million was associated with a small but significant decrease in ICECAP-A utility of − 0.0087 (95% CI: − 0.014, − 0.04). Across all examined sub-groups defined by age, gender, and education, these results were practically the same, as none of the included interactions reached significance and the coefficients of the reference groups were not significantly different from the overall estimates (columns 2–4). Somewhat counterintuitive results were found for the associations between different levels of health and the variables of interest: the largest negative association between COVID-19 mortality and well-being was found for individuals in good health (− 0.0053), while the association was positive compared to the reference group and the overall effect (0.0161) for individuals with more severe health problems. Concurrently, stringency and incidence rate were positively associated with well-being in individuals in good health, while stringency was negatively correlated with well-being for individuals with more severe health problems (− 0.0089). A possible explanation behind these diverging patterns may relate to the vastly different base line levels of well-being across health levels (see Fig. 7).

**Table 1:** Associations of incidence, mortality, and stringency with ICECAP-A utility score

	Overall	Age	Gender	Education	Health	Income
Increase in 7-day incidence by 100 per 100 k	− 0.0000	0.0001	− 0.0000	− 0.0001	− 0.0002	0.0001
Increase in 7-day mortality by 10 per million	− 0.0087^*^	− 0.0089^*^	− 0.0080^*^	− 0.0102^*^	− 0.0089^*^	− 0.0067^*^
Increase in Stringency Index by 10 points	0.0004	0.0004	0.0003	0.0005	− 0.0000	0.0010
Incidence × Age < 35		− 0.0003				
Mortality × Age < 35		− 0.0016				
Stringency × Age < 35		− 0.0003				
Incidence × Age ≥ 65		− 0.0002				
Mortality × Age ≥ 65		0.0033				
Stringency × Age ≥ 65		− 0.0001				
Incidence × Female			− 0.0000			
Mortality × Female			− 0.0015			
Stringency × Female			0.0000			
Incidence × Tertiary educated				0.0000		
Mortality × Tertiary educated				0.0032		
Stringency × Tertiary educated				− 0.0003		
Incidence × No health problems^2^					0.0006^*^	
Mortality × No health problems					− 0.0053^+^	
Stringency × No health problems					0.0056^*^	
Incidence × More severe health problems^1^					− 0.0002	
Mortality × More severe health problems					0.0161^*^	
Stringency × More severe health problems					− 0.0089^*^	
Incidence × Financial insecure^3^						− 0.0003
Mortality × Financial insecure						− 0.0047
Stringency × Financial insecure						− 0.0016^*^
Observations	64,046	64,046	64,046	64,046	64,046	64,046
Individuals	24,992	24,992	24,992	24,992	24,992	24,992

Results from individual fixed regression, controlling for wave fixed effects. ^1^EQ-5D sum score = 5, ^2^EQ-5D sum score ≥ 10, ^3^Having some or great difficulties with making ends meet. Reference groups are age between 35 and 64, male, non-tertiary educated, some health problems (EQ-5D sum score between 6 and 9), financial secure. ^+^*p* < 0.10, *p < 0.05

There was a negative and significant association between stringency index and well-being in individuals with a financially less secure situation compared to more financially stable individuals (− 0.0016).

Results from the separate regression for the different ICECAP-A dimensions are shown in Table 2. Incidence rate and stringency index were not significantly correlated with any of the ICECAP-A dimensions. The largest significant association with COVID-19 mortality was found for the stability (− 0.0154), achievement (− 0.0122), and autonomy (− 0.0115) dimensions, while mortality was not significantly associated with the attachment dimension.

**Table 2:** Associations of incidence, mortality, and stringency with ICECAP-A dimensions

	Utility	Stability	Attachment	Autonomy	Achievement	Enjoyment
Increase in 7-day incidence by 100 per 100 k	− 0.0000	− 0.0002	0.0000	− 0.0001	0.0001	− 0.0004
Increase in 7-day mortality by 10 per million	− 0.0087^*^	− 0.0154^*^	− 0.0008	− 0.0115^*^	− 0.0122^*^	− 0.0073^+^
Increase in stringency index by 10 points	0.0004	0.0009	− 0.0007	0.0025^+^	− 0.0010	− 0.0020
Observations	64,046	64,046	64,046	64,046	64,046	64,046
Individuals	24,992	24,992	24,992	24,992	24,992	24,992

Results from individual fixed regression, controlling for wave fixed effects. ICECAP-A dimensions were rescaled to a 0 to 1 range. ^+^*p* < 0.10, *p < 0.05

Appendix 4 contains the corresponding results for the associations across sub-groups, which are summarized in the following: Most notable results for the **stability** dimension are that the significant association with mortality (− 0.0154) almost doubled in individuals aged below 35 (combined association: − 0.029) and that the negative association with stringency was again highest in individuals in a financially less secure situation (combined association: − 0.020). No noteworthy sub-group results were found in the **attachment** dimension. Stringency was positively correlated with the **autonomy** dimension, especially in financially stable individuals (+ 0.0034). The largest negative associations between mortality and **achievement** were found for individuals with a tertiary education (− 0.0165), and individuals with no health problems (combined association: − 0.0256). Results from the regression on the **enjoyment** dimension showed that the negative associations between mortality and enjoyment were highest in males and individuals with no health problems. Neither incidence, mortality, nor stringency were significantly negatively correlated with enjoyment in individuals with higher financial security. Larger negative associations of mortality with capabilities in healthier individuals, and positive associations with capabilities in sicker individuals (compared to relatively healthy individuals), as well as more negative associations of stringency with capabilities in sicker individuals were found consistently across ICECAP-A dimensions.

## Discussion

### Summary of findings

This exploratory study aimed to provide insight into well-being patterns over the course of the COVID-19 pandemic in Europe, using a multi-dimensional capability well-being measure (the ICECAP-A).A first general finding is that capability well-being changes, overall and in the underlying dimensions, varied considerably between countries. Two somewhat deviating overall trends were identified. Denmark, the Netherlands, and France experienced a u-shaped pattern, i.e., a drop in capability well-being (highest in France) over autumn and winter 2020/21, with a recovery until June 2021. However, in the UK, Germany, Portugal, and Italy, capability well-being rather followed an m-shape. This entailed a recovery of well-being levels in June 2020 compared to April 2020, a drop in autumn and winter (largest in Germany and Italy), a recovery over the summer 2021, and a steep decline in autumn 2021. At this point, it is important to note that it is difficult to assess how meaningful the observed overall within country changes (ranging up to ± 0.04 on a 0 to 1 scale) are as comparable longitudinal data or estimates for a minimally important difference for the ICECAP-A are not (yet) available. However, values from the ICECAP-A literature can give an indication. For instance, in a population of adults with knee pain, a minimally important decrease in EQ-5D-3L utility over a 6-month period coincided with a mean decrease in ICECAP-A score of − 0.054 (95% CI: − 0.084, − 0.024). A multi-country study of English speaking countries reported ICECAP-A differences between healthy individuals (ICECAP-A mean: 0.89) and individuals with hearing loss (0.85), heart disease (0.82), mild depression (0.78), and moderate arthritis (0.82) [28]. The overall within country mean ICECAP-A changes observed in this study between summer 2020 and winter 2021 of up to − 0.037 in France and − 0.041 in Germany, as well as some changes observed in the sub-groups (e.g., individuals age 35 and younger in the UK with a change of − 0.093) are similar to these reported differences in ICECAP-A scores. As such, with the exception for Denmark (− 0.02), the observed overall decreases in capability well-being in the winter of 2020/2021 can be argued to represent small but meaningful differences.

Examining the five dimensions of the ICECAP-A revealed that compared to April 2020, the stability dimension generally increased in the countries that were most severely affected by the first COVID-19 wave. Longer term decreases were predominantly found for the attachment dimension, with Italy and France being most affected, and the enjoyment dimension, which decreased in all countries in the winter of 2020/21. Lowest levels of capability well-being were found in the youngest age group, for individuals in worse health, and individuals who had difficulties making ends meet. We found only small differences between gender and education levels.

The regression analysis revealed that COVID-19 mortality was consistently negatively correlated with capability well-being and its sub-dimensions, while stringency, with the exception of a positive correlation with autonomy, and incidence rate were generally not significantly associated with well-being. At the same time, the calculated correlations between COVID-19 mortality and ICECAP-A were rather small, with the most negative coefficients found in the stability dimension, specifically in the youngest age group. Across well-being dimensions, negative associations of well-being and COVID-19 mortality, and also partly stringency, were highest in individuals with a less stable financial situation, while associations in financially stable individuals were often non-significant. Noteworthy is that individuals in worse health appeared to be less impacted by COVID-19-related variables, and that stringency was even positively associated with autonomy and stability in financially stable individuals.

### Strengths and weaknesses of this study

This study has several notable strengths. First, we were able to use unique, high-frequency well-being data spanning over nine time points from April 2020 to January 2022. Second, the data included seven European countries, which differed regarding the severity of COVID-19 and the timing and stringency of containment policies (Fig. 1). Third, contrary to the existing literature, a multi-dimensional measure for well-being was used, allowing for a more nuanced analysis of well-being patterns. Therefore, this study provides the most comprehensive and detailed overview to date of well-being patterns in Europe over the course of the COVID-19 pandemic.

Nevertheless, the study also has a few noteworthy limitations. First, we were confined to analyzing a specific type of well-being, namely capability well-being, which somewhat limits the comparison of our findings to those from other studies. This capability approach to well-being underlying the ICECAP-A is conceptually different from the subjective well-being measures more frequently used in the related literature. However, the use of capability well-being as an outcome measure in health economic evaluations is gaining in importance [31]. Furthermore, measuring capabilities, i.e., what one can do and be, may be considered more relevant in the pandemic context than subjective well-being, as the risk of infection and the government restrictions precisely infringed people’s possibilities or freedoms to do or be. At the same time, to construct the overall capability well-being measure, we had to use weights, which were derived for the UK, as such weights were not available for other countries (except for the Netherlands) [33]. This may have introduced some imprecision, since preferences for the different dimensions likely differ across countries, as was observed for the EQ-5D before [36]. The presented patterns at dimension level, however, do not have this issue. A second major limitation is that no pre-pandemic reference data for the ICECAP-A were available, as the ECOS survey was initiated in response to the first COVID-19 wave and lockdowns in Europe. Lacking pre-pandemic longitudinal ICECAP-A data, we cannot rule out that parts of the changes in capability well-being were influenced by seasonal patterns. For instance, small changes in happiness from the 3rd to the 4th quarter have regularly been observed in the UK before the pandemic [37]. Whether this also holds for capability well-being is unclear. Moreover, COVID-19 incidence and the corresponding measures also closely follow a seasonal pattern, which would complicate disentangling a direct seasonal effect from this indirect effect.

The exploratory nature of this study also implies that none of the presented regression results should be interpreted as causal effects. Designing a valid quasi-experimental study appears to be worthwhile to also disentangle the causal mechanisms of stringency, mortality, and well-being. However, this is outside the scope of this paper and is complicated by the mostly concurrent timing of COVID-19 waves and the corresponding governmental measures across countries in Europe.

### Findings in relation to previous studies

Our study generally confirms findings on negative well-being changes in Europe during the COVID-19 pandemic [3], especially in times with high incidence and mortality rates and more stringent governmental measures [7, 35, 38, 39]. At the same time, this study also confirmed that these changes on average were often small, with the exception of changes between summer 2020 and winter 2020/2021, but also highly heterogeneous [8]. The overall caveat of comparisons with the broader well-being literature, though, is that most previous studies were limited to the first phase of the pandemic in Europe. However, longitudinal evidence on mental health trajectories also suggests only small negative changes, also across different European countries [40, 41]. Yearly life satisfaction data from the World Happiness report also showed no considerable negative changes in most European countries, comparing averages from the years 2021 and 2020 with 2019 data [9, 42]. Comparable data on capability well-being measured using the ICECAP do not exist. However, a recent study attempted to approximate the capability well-being impact of the initial lockdown through a cross-sectional survey with two recall timepoints [43]. Given the largest changes in average level of capability well-being we observed (0.04 for Italy and Germany), their findings of a 0.05 decrease in the Netherlands, and especially, of a 0.10 decrease in the UK appear to be overestimates.

In terms of relevant sub-group results, our analysis confirmed that younger age groups experienced larger negative well-being changes during the pandemic [12], which was also found before for mental health [10, 11]. While there is some evidence that the oldest old may have been severely impacted by government measures like visitor bans to long-term care homes [44], the available data may have a blind spot in this demographic since this specific group is generally underrepresented in online surveys. At the same time, older individuals were likely less impacted by other measures and their wider impact, for instance relating to home schooling, home office, or financial threats due to unemployment or a general economic downturn.

Finding that females and males share similar well-being patterns, with females not experiencing larger well-being changes, is at odds with results from previous literature, which, however, focused on mental health differences [12, 15]. While previous studies showed that mental health issues were more prevalent among females, this question is less clear cut for well-being [45, 46]. In terms of the role of income and health, the results of our study are in line with previous evidence regarding the role of problems with income [14, 20] and poor health [11] for well-being, and in particular, capability well-being [47], during the COVID-19 pandemic.

### Implication of results

A broader conclusion that can be drawn from the presented results is that capability well-being changes at the population level over the course of the pandemic mostly appear to be fairly modest. While certain sub-groups suffered larger, meaningful decreases, it appears that populations in general were fairly resilient in the face of the adversities caused by COVID-19 and related government measures. The well-being reductions observed in the winter of 2020/2021 were mostly made up for before the summer of 2021. This implies that populations rather quickly adapted to changing circumstances. Concurrently, it has been shown that the impact of high COVID-19 mortality rates on anxiety and depression is moderated by the subsequent stringent governmental measures, confirming what the authors call a ‘welcomed lockdown’ hypothesis [48]. To what extent these findings also relate to the merely moderate observed changes in well-being is unclear, however this phenomenon might partly explain some of the more counterintuitive associations in certain sub-groups.

The presented overall well-being patterns also do not suggest a general downward trend in well-being across the pandemic (with the possible exception of France, Fig. 2). While it is too early for a final verdict, this hints toward the possibility that the pandemic may not leave a permanent mark on well-being levels in Europe.

Although this study is exploratory in nature, it can be observed that the timing of reductions in capability well-being coincided with higher COVID-19 incidence and mortality rates and more stringent containment measures by governments. At the same time, while the pandemic developed fairly similarly across the included countries, changes in capability well-being in the overall populations as well as in sub-groups differed considerably across countries. Objectively similar containment measures might, thus, have had a differential impact on capability well-being due to differences in health and social care systems, lifestyle and culture across Europe. The finding that the largest well-being decreases were observed in the enjoyment dimension may be explained by several factors. Social distancing measures (whether imposed or self-imposed), in particular closures of public spaces, cancelation of events, and not being able to engage in various social and sport activities, infringed on people’s capability to do and to be things in life that matter to them.

In terms of future research agendas, this study could be a starting point for investigating causes and mechanisms for the different well-being patterns observed over the course of the pandemic, especially across sub-groups. It seems worthwhile attempting to disentangle the well-being impacts of COVID-19 incidence and mortality on the one hand, and containment measures on the other hand, in particular also across age groups. Such efforts will likely entail examining specific policies and their impact using quasi-experimental approaches based on other observational datasets. A policy recommendation for future COVID-19 waves or other future pandemics is that imposing stringent measures on citizens should be accompanied by monitoring not only effects on health but also on well-being in society. These efforts should be particularly targeted at the sub-groups in the population that have experienced the largest declines in the first one and half year of the COVID-19 pandemic (e.g., younger age groups, and less affluent and less healthy individuals). Once evidence on the differential impact of certain policies on sub-populations becomes available, more informed decisions can be made when imposing new measures or setting up support mechanisms.

## Conclusion

We investigated changes in capability well-being over the course of the COVID-19 pandemic across seven European countries and nine time points spanning from April 2020 to January 2022. We found notable difference in absolute well-being levels across countries, but the general patterns, with meaningfully lower levels observed in winter 2020/21 and a recovery in summer 2021, were similar across countries with no clear evidence for a general downward trend in well-being. Observed well-being reductions were small on average, but heterogenous across sub-groups, showing the importance of keeping in mind the welfare of the most vulnerable during an extraordinary event like the COVID-19 pandemic.

### Supplementary Information

Below is the link to the electronic supplementary material.

Supplementary file1 (DOCX 542 KB)

## Data Availability

The ECOS data are currently not shared with third parties but can be made available upon reasonable request. Data requests can be directed to the data access committee of our COVID-19 Survey (contact via jonas.schreyoegg@uni-hamburg.de or info@hche.de). Code for the analysis available upon request.

## Declarations

### Transparency statement

The lead author (S.H.) affirms that this manuscript is an honest, accurate, and transparent account of the study being reported; that no important aspects of the study have been omitted; and that any discrepancies from the study as planned (and, if relevant, registered) have been explained.
