# Correction: Braving the waves: exploring capability well-being patterns in seven European countries during the COVID-19 pandemic

**DOI:** 10.1007/s10198-023-01636-0

**Published:** 2023-10-21

**Authors:** Sebastian Himmler, Job van Exel, Werner Brouwer, Sebastian Neumann-Böhme, Iryna Sabat, Jonas Schreyögg, Tom Stargardt, Pedro Pita Barros, Aleksandra Torbica

**Affiliations:** 1 Erasmus School of Health Policy and Management, Erasmus University Rotterdam, P.O. Box 1738, 3000 DR Rotterdam, The Netherlands; 2 Erasmus Centre for Health Economics Research (EsCHER), Erasmus University Rotterdam, Rotterdam, The Netherlands; 3 Hamburg Center for Health Economics, University of Hamburg, Esplanade 36, 20354 Hamburg, Germany; 4 Nova School of Business and Economics, R. Holanda 1, 2775-405 Carcavelos, Portugal; 5 Centre for Research On Health and Social Care Management, CERGAS, Bocconi University, Via Röntgen N. 1, 20136 Milan, Italy

**Correction: The European Journal of Health Economics** 10.1007/s10198-023-01604-8

In this article the author name Neumann-Böhme was incorrectly written as Neuman-Böhme.

The original article has been udpated.

