# Supplementary material for: Braving the waves: exploring capability well-being patterns in seven European countries during the COVID-19 pandemic
Source: Eur J Health Econ. 2023 Jul 6;25(4):563–78. doi: 10.1007/s10198-023-01604-8 (PMC11136820; doi:10.1007/s10198-023-01604-8)
Supplement: Supplementary file 1 — Supplementary file1 (DOCX 542 KB) [file 10198_2023_1604_MOESM1_ESM.docx]

# Braving the waves – A descriptive study of capability well-being patterns in seven European countries during the COVID-19 pandemic

#

Online supplementary materials

**Appendix 1**

**Education categorization for the seven included countries**

**Table A1** Education categorisation

| UK | | 1 | | Combined Junior and Infant School/ Infant School | | Low | |
| --- | --- | --- | --- | --- | --- | --- | --- |
|  |  | 2 | | Junior School | |  |  |
|  |  | 3 | | Comprehensive School | |  |  |
|  |  | 4 | | Comprehensive School (GCSE)/ Secondary Modern (GCSE)/ Grammar School (GSCE)/ City Technology College (CGSE)/ Sixth Form | | Middle | |
|  |  | 5 | | College and Institution of Higher education | | High | |
|  |  | 6 | | Open College -College of Technology - Institute/ Teacher Training College | |  |  |
|  |  | 7 | | University/ Open University | |  |  |
| Portugal | | 1 | | Sem Estudos | | Low | |
|  |  | 2 | | Primário Incompleto | |  |  |
|  |  | 3 | | Primário Completo | |  |  |
|  |  | 4 | | Nível Médio Incompleto | | Middle | |
|  |  | 5 | | Nível Médio Completo | |  |  |
|  |  | 6 | | Superior Incompleto | | High | |
|  |  | 7 | | Superior Completo | |  |  |
| Netherlands | 1 | | LO (lagere school, LAVO, VGLO) | | Low | |  |
|  | 2 | | LBO (LBO, LTS, ITO, LEAO, Huishoudschool, LLO) | |  |  |  |
|  | 3 | | MAO (MAVO, IVO, MULO, ULO, 3jr HBS, 3jr VWO, 3jr VHMO) | |  |  |  |
|  | 4 | | MBO (MTS, UTS, MEAO) | | Middle | |  |
|  | 5 | | HAO (HAVO, VWO, Atheneum, Gymnasium, NMS, HBS, Lyceum) | |  |  |  |
|  | 6 | | HBO (HTS, HEAO, Wetensch. kand., Univers. onderwijs kand.) | | High | |  |
|  | 7 | | WO (Universitair onderwijs, Doctoraalopleiding, TH) | |  |  |  |
| Italy | | 1 | | scuola elementare | | Low | |
|  |  | 2 | | scuola media inferiore | |  |  |
|  |  | 3 | | istituto professionale | | Middle | |
|  |  | 4 | | scuola superiore | |  |  |
|  |  | 5 | | università | | High | |
|  |  | 6 | | master | |  |  |
|  |  | 7 | | dottorato | |  |  |

**Table A1** continued.

| Germany | 1 | Grundschule | Low |
| --- | --- | --- | --- |
|  | 2 | Hauptschule |  |
|  | 3 | Realschule |  |
|  | 4 | Gymnasium/ Berufliches Gymnasium/ Fachgymnasium, Gesamtschule | Middle |
|  | 5 | Fachoberschule, Fachschule, Berufsschule, Berufsfachschule |  |
|  | 6 | Technische Hochschule, Pädagogische Hochschule, Kunsthochschule/ Musikhochschule | High |
|  | 7 | Fachhochschule |  |
|  | 8 | Universität, Technische Universität |  |
| France | 1 | École Primaire | Low |
|  | 2 | Collège |  |
|  | 3 | Lycée d`Enseignement général et technologique | Middle |
|  | 4 | Lycée professionnel |  |
|  | 5 | Grande École de Commerce et de Gestion/ scientifique, Établissement d`Enseignement supérieur catholique/ artistique/ agricole | High |
|  | 6 | Grand Établissement/ École normale supérieure/ d`Ingénieur/ d`Architecture/ nationale vétérinaire |  |
|  | 7 | Université, Institut universitaire de Technologie/ national polytechnique/ d`Études politiques/ universitaire de Formation des Maîtres |  |
| Denmark | 1 | Folkeskolen - f.eks. 9. eller 10. klasse | Low |
|  | 2 | Gymnasial uddannelse - f.eks. Almen Gymnasium, HHX, HTX osv. | Middle |
|  | 3 | En videregående erhvervsuddannelse - f.eks. landbrugs-, social- og sundheds uddannelser, produktionsskole | High |
|  | 4 | En mellemlang videregående uddannelse |  |
|  | 5 | Universitets uddannelse |  |

#

**Appendix 2**

**Table A2** Demographic characteristics of country samples – proportions and means (SD).

|  | **UK** | **PT** | **NL** | **IT** | **GER** | **FR** | **DK** | **Total** |
| --- | --- | --- | --- | --- | --- | --- | --- | --- |
| Female | 0.51 | 0.51 | 0.52 | 0.52 | 0.51 | 0.53 | 0.52 | 0.52 |
| **Age** |  |  |  |  |  |  |  |  |
| Age in years | 49.3 (17.2) | 44.0 (15.1) | 48.6 (16.5) | 48.9 (16.3) | 49.9 (16.3) | 48.2 (16.5) | 48.9 (16.5) | 48.3 (16.5) |
| <35 | 0.26 | 0.34 | 0.26 | 0.25 | 0.24 | 0.27 | 0.25 | 0.27 |
| 35-64 | 0.5 | 0.54 | 0.53 | 0.51 | 0.51 | 0.5 | 0.52 | 0.52 |
| 65+ | 0.24 | 0.12 | 0.21 | 0.24 | 0.26 | 0.22 | 0.23 | 0.22 |
| **Education** |  |  |  |  |  |  |  |  |
| Low educ | 0.14 | 0.12 | 0.28 | 0.26 | 0.19 | 0.14 | 0.13 | 0.18 |
| Med educ | 0.39 | 0.38 | 0.41 | 0.51 | 0.52 | 0.5 | 0.33 | 0.43 |
| High educ | 0.47 | 0.5 | 0.31 | 0.23 | 0.3 | 0.36 | 0.54 | 0.39 |
| **Financial security** |  |  |  |  |  |  |  |  |
| Insecure | 0.37 | 0.34 | 0.41 | 0.58 | 0.44 | 0.55 | 0.38 | 0.44 |
| Secure | 0.63 | 0.66 | 0.59 | 0.42 | 0.56 | 0.45 | 0.62 | 0.56 |
| **Health** |  |  |  |  |  |  |  |  |
| EQ-5D-5L^*^ | 7.87 (3.54) | 6.76 (2.17) | 7.47 (3.07) | 7.10 (2.63) | 7.91 (3.40) | 7.44 (3.04) | 7.91 (3.29) | 7.49 (3.08) |
| Low health | 0.24 | 0.096 | 0.21 | 0.14 | 0.25 | 0.18 | 0.25 | 0.19 |
| Healthy | 0.44 | 0.57 | 0.43 | 0.53 | 0.44 | 0.49 | 0.46 | 0.48 |
| Full health | 0.32 | 0.33 | 0.36 | 0.33 | 0.31 | 0.33 | 0.29 | 0.32 |
| Observations | 9181 | 9214 | 9129 | 9238 | 9170 | 9260 | 9111 | 64303 |

SD=standard deviation; EQ-5D-5L=European Quality of Life 5 Dimensions 5 Level Version
^*^Sum score from 5 (full health) to 25.

**Table A3** ICECAP dimension scores and overall capability well-being in wave 1 (April 2020).

|  | **Stability** | | **Attachement** | | **Autonomy** | | **Achievement** | | **Enjoyment** | | **Capability well-being** | | **N** |
| --- | --- | --- | --- | --- | --- | --- | --- | --- | --- | --- | --- | --- | --- |
|  | Mean | SD | Mean | SD | Mean | SD | Mean | SD | Mean | SD | Mean | SD |  |
| **UK** | 2.84 | 0.85 | 3.11 | 0.83 | 3.30 | 0.77 | 2.85 | 0.79 | 3.00 | 0.79 | 0.78 | 0.19 | 1009 |
| **PT** | 2.70 | 0.80 | 3.15 | 0.78 | 3.23 | 0.74 | 2.76 | 0.71 | 2.72 | 0.79 | 0.75 | 0.18 | 1064 |
| **NL** | 3.16 | 0.70 | 3.20 | 0.75 | 3.27 | 0.71 | 2.87 | 0.75 | 3.13 | 0.77 | 0.82 | 0.15 | 1012 |
| **IT** | 2.53 | 0.90 | 3.12 | 0.81 | 3.07 | 0.89 | 2.74 | 0.81 | 2.86 | 0.80 | 0.73 | 0.20 | 1077 |
| **GER** | 2.96 | 0.77 | 3.00 | 0.82 | 3.08 | 0.76 | 2.74 | 0.79 | 2.77 | 0.76 | 0.75 | 0.18 | 1002 |
| **FR** | 2.95 | 0.70 | 3.19 | 0.76 | 3.25 | 0.73 | 2.91 | 0.71 | 3.16 | 0.76 | 0.81 | 0.16 | 1000 |
| **DK** | 3.18 | 0.69 | 3.13 | 0.80 | 3.26 | 0.71 | 3.01 | 0.72 | 3.28 | 0.73 | 0.83 | 0.17 | 1000 |

Note: N, number of observations. Dimension scores range from 1 (worst) to 4 (best).


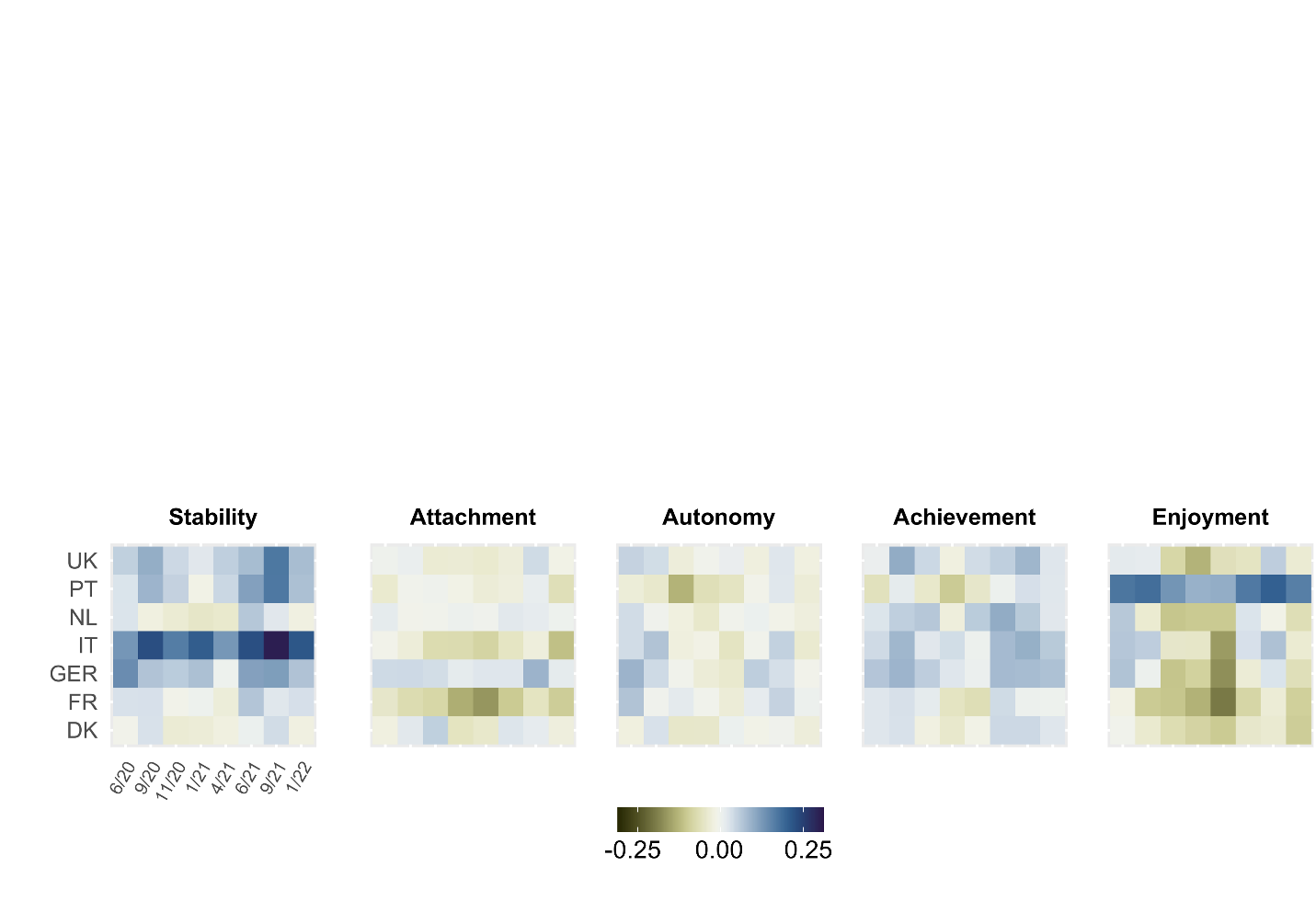


**Figure A 1** Difference in mean score per well-being dimension compared to 1^st^ wave (April 2020) across countries and waves of data collection. ICECAP-A dimension scores range from 1 (worst) to 4 (best). Number of observations per cell ~1,000.

Regarding the stability dimension, there was large variation in differences across countries as compared to April 2020. While there was a general upward trend in Italy over time (e.g., +0.30 in September 2021), there were also temporal declines in scores on stability in the Winter of 2020/21, most pronounced in countries like the Netherlands (up to -0.05) and Denmark (up to -0.03). Largest declines in scores on the attachment dimension were observed in France (up to -0.16) and Italy (up to -0.11), mostly concentrated in the Winter and spring 2021. Other countries did not experience a noteworthy decline in attachment as compared to April 2020. Negative changes in scores on the autonomy dimension could be observed for all countries for autumn 2020 and winter 2021, while the largest decline was found for Portugal (-0.13 in November 2020). Reductions in the achievement dimension were observed in Portugal (-0.10), France (-0.07) and Denmark (-0.04) in autumn 2020 and winter 2021. The overall largest declines in well-being were found in the enjoyment dimension. While for Portugal changes were still positive as compared to the very low score observed in April 2020 (2.72, SD=0.79), enjoyment notably declined with the start of autumn 2020, with the largest and longest declines observed in France (up to -0.20) and Germany (up to -0.17).

**Appendix 3**

**Changes in well-being dimensions across subgroups**

**Age groups**

**
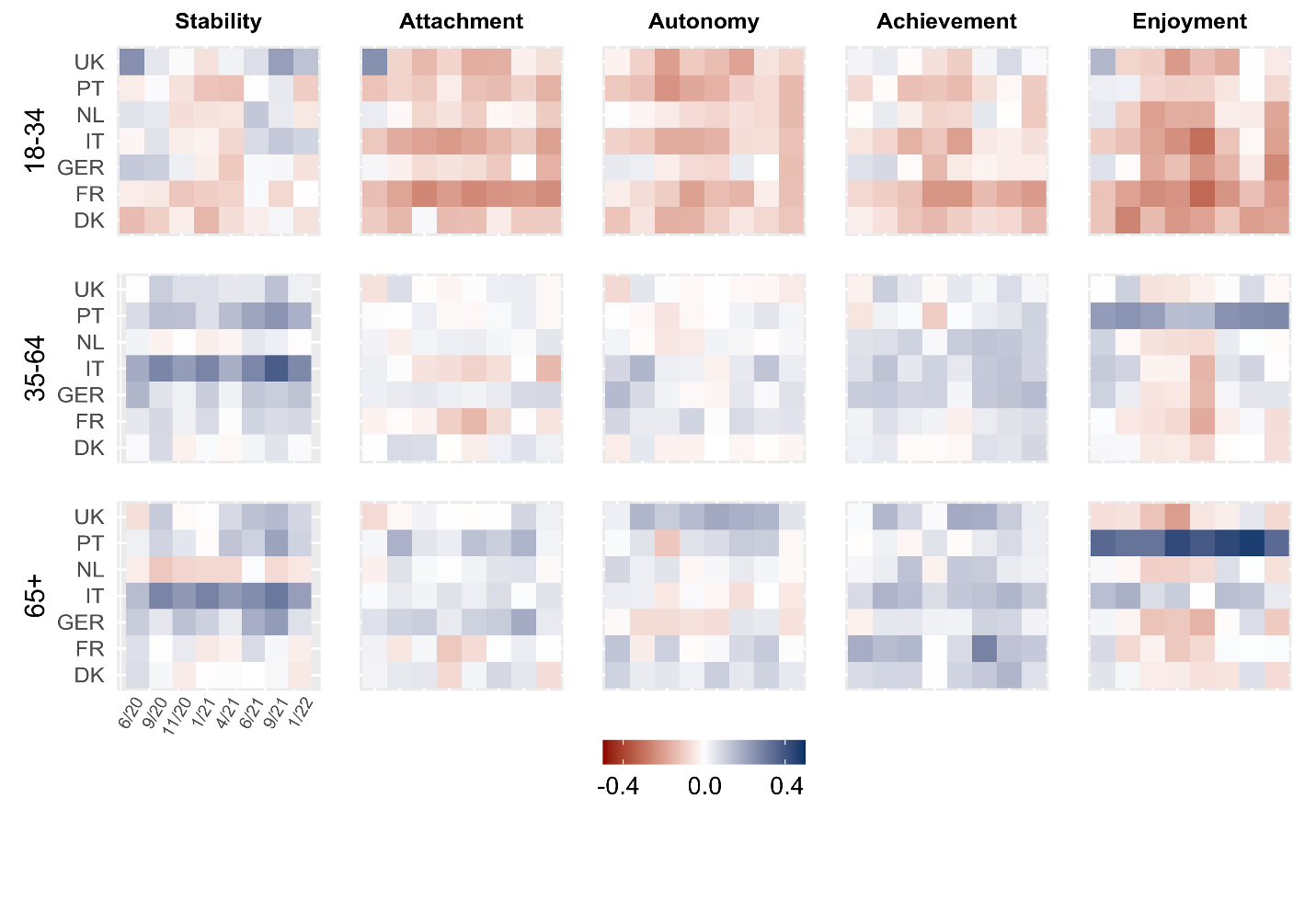
**

**Fig A 2** Difference in mean ICECAP-A dimension scores compared to April 2020 across age groups. Mean number of observations per cell for the three age groups is 272 (18-34), 526 (35-64), and 222 (65+).

**Gender**

**
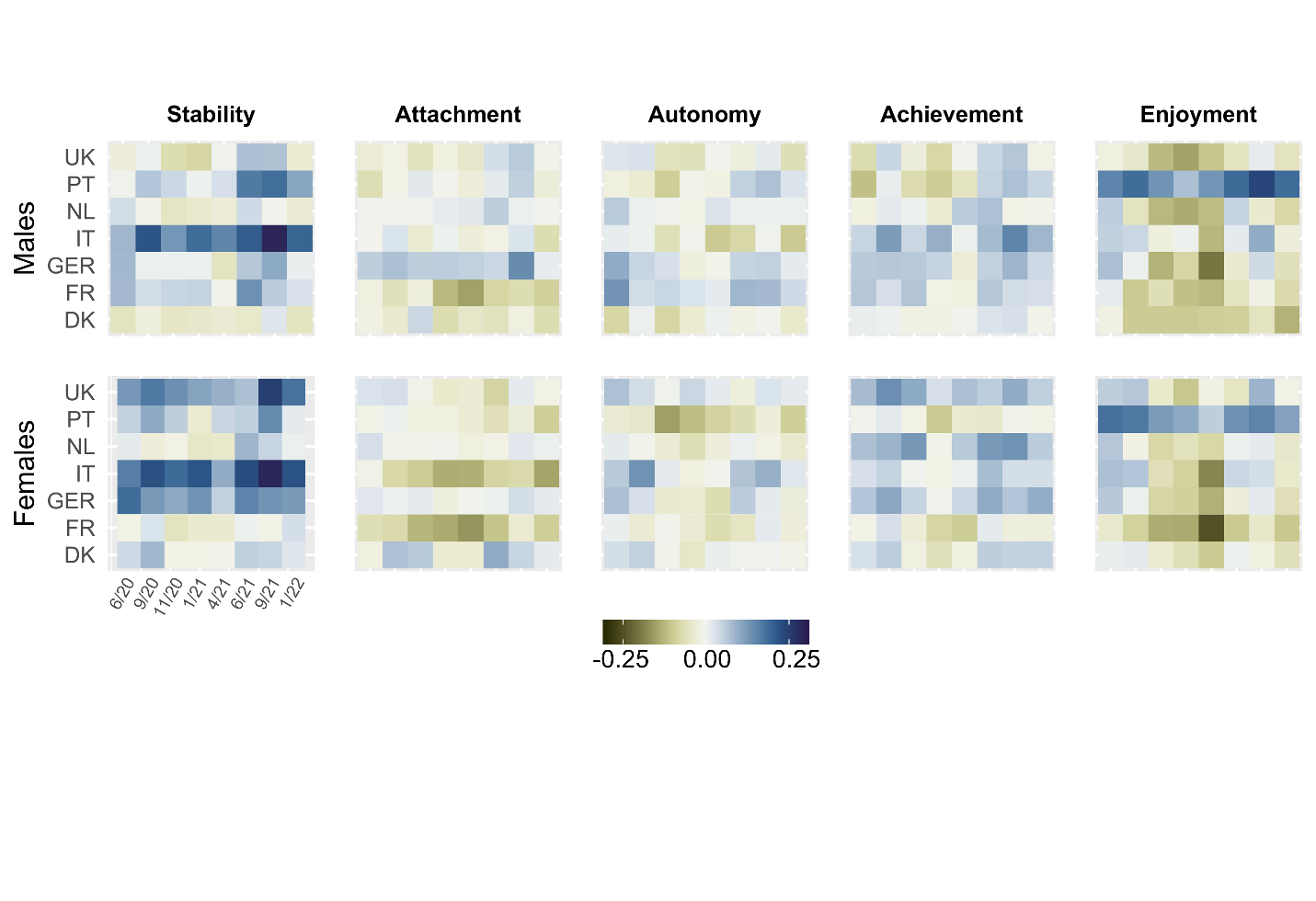
**

**Fig A 3** Difference in mean ICECAP-A dimension scores compared to April 2020 across gender. Mean number of observations per cell for the two groups is 530 (female), and 491 (male).

**Education**

**
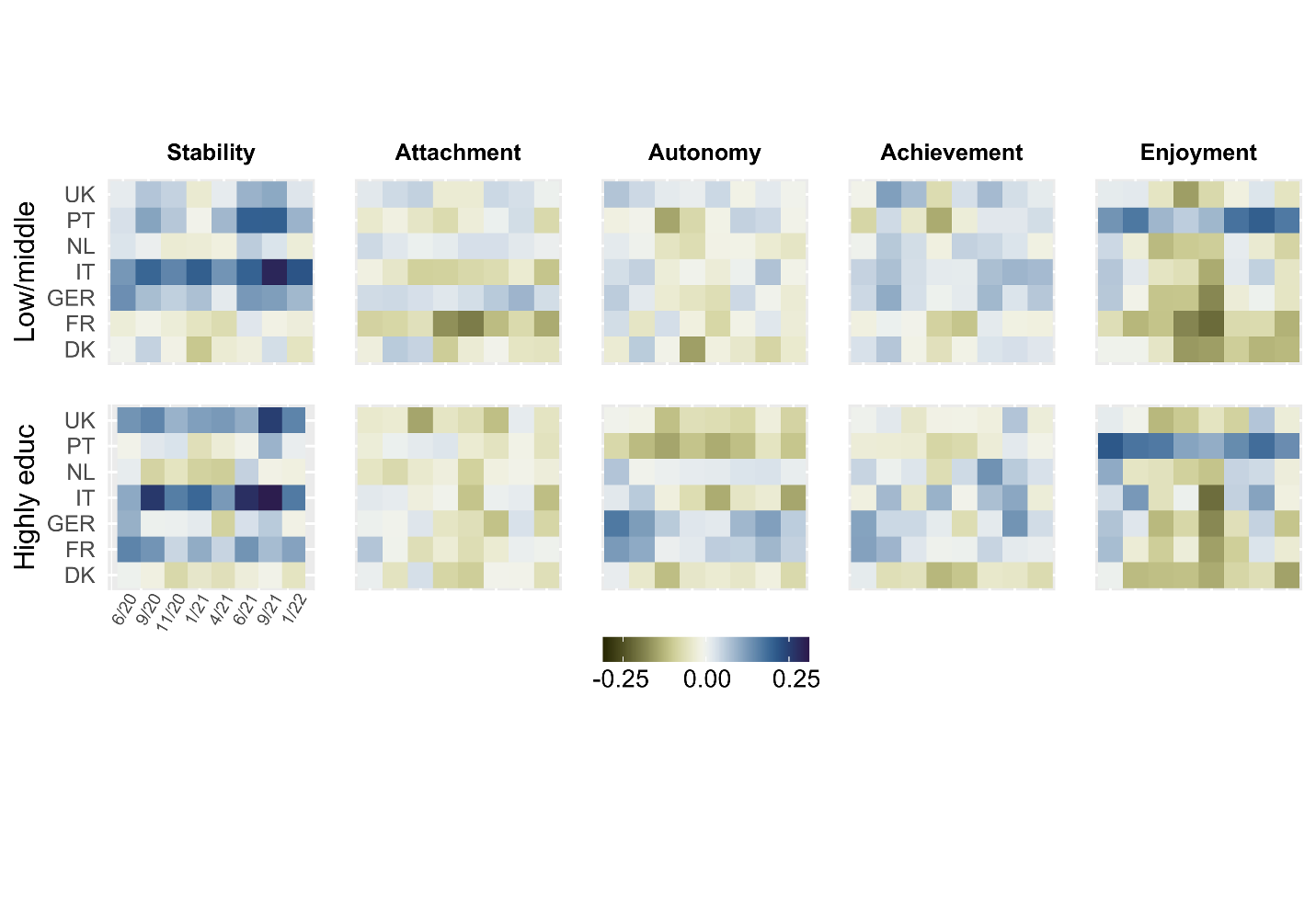
**

**Fig A 4** Difference in mean ICECAP-A dimension scores compared to April 2020 across education levels. Mean number of observations per cell for the two education groups is 617 (Low & middle) and 387 (Highly).

**Health status
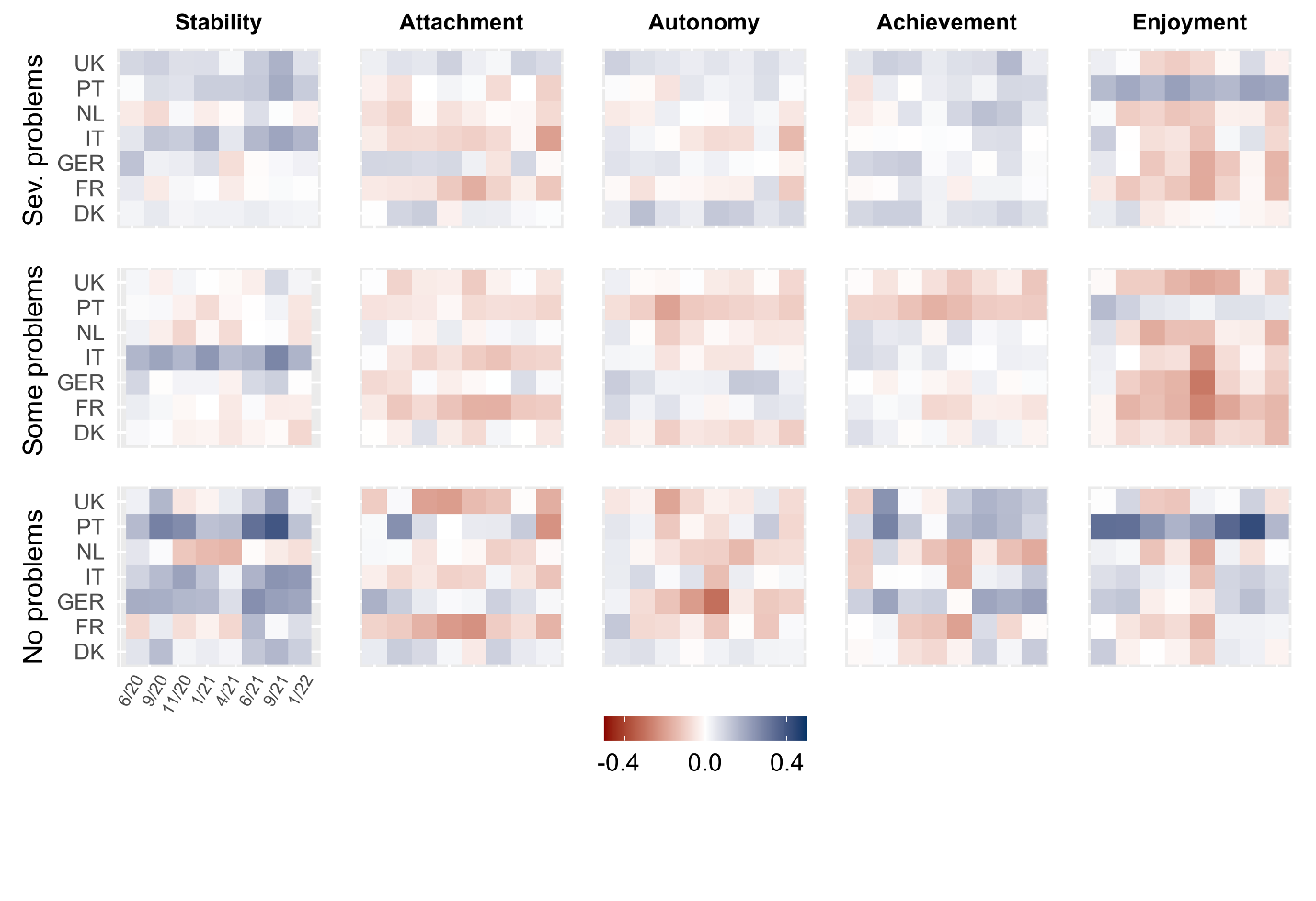
**

**Fig A 5** Difference in mean ICECAP-A dimension scores compared to April 2020 differentiated by health status measured using EQ-5D sum score. Full health (5), Healthy (6-9), Lower health (10+) on a theoretical range of 5 to 25. Mean number of observations per cell for the three groups is 331 (Full health), 492 (Healthy), and 198 (Lower health).

**Financial security
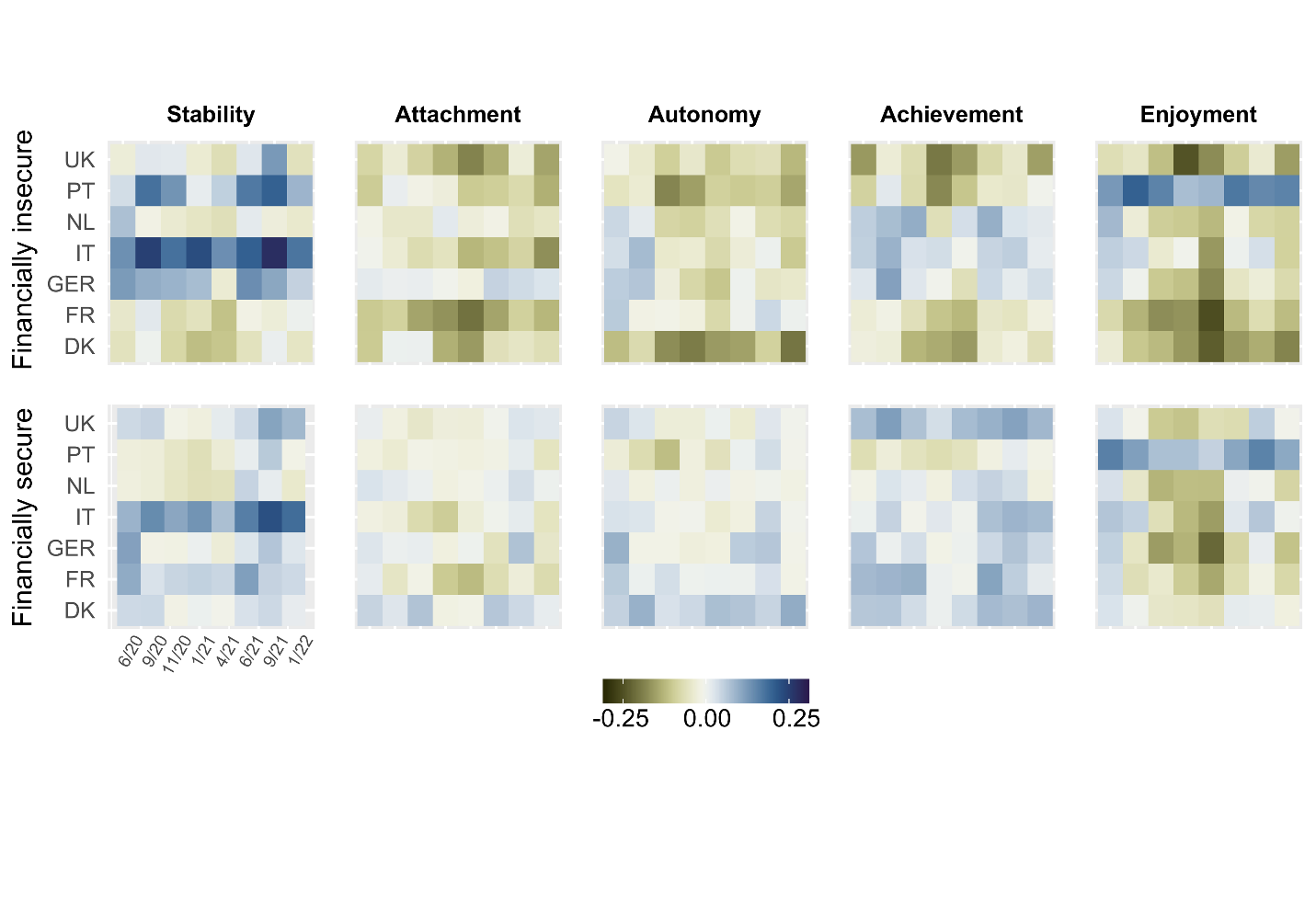
**

**Fig A 6** Difference in mean ICECAP-A dimension scores (1 lowest, 4 highest) compared to April 2020 differentiated by financial situation. “Unstable” defined as having some or great difficulties with making ends meet. Mean number of observations per cell for the two groups is 448 (secure), and 573 (insecure).

**Appendix 4**

**Regression results for ICECAP-A dimensions**

**Table A4** Associations of incidence, mortality and stringency with the ICECAP-A stability dimension.

|  | Overall | Age | Gender | Education | Health | Income |
| --- | --- | --- | --- | --- | --- | --- |
| Increase in 7-day incidence by 100 per 100k | -0.0002 | -0.0002 | -0.0004 | -0.0002 | -0.0007^*^ | -0.0001 |
| Increase in 7-day mortality by 10 per million | -0.0154^*^ | -0.0139^*^ | -0.0170^*^ | -0.0143^*^ | -0.0139^*^ | -0.0163^*^ |
| Increase in Stringency Index by 10 points | 0.0009 | 0.0004 | 0.0005 | 0.0007 | -0.0005 | 0.0024^+^ |
| Incidence × Age < 35 |  | 0.0008^+^ |  |  |  |  |
| Mortality × Age < 35 |  | -0.0130^+^ |  |  |  |  |
| Stringency × Age < 35 |  | 0.0004 |  |  |  |  |
| Incidence × Age >= 65 |  | -0.0005 |  |  |  |  |
| Mortality × Age >= 65 |  | 0.0089 |  |  |  |  |
| Stringency × Age >= 65 |  | 0.0010 |  |  |  |  |
| Incidence × Female |  |  | 0.0004 |  |  |  |
| Mortality × Female |  |  | 0.0033 |  |  |  |
| Stringency × Female |  |  | 0.0008 |  |  |  |
| Incidence × Tertiary educated |  |  |  | 0.0001 |  |  |
| Mortality × Tertiary educated |  |  |  | -0.0026 |  |  |
| Stringency × Tertiary educated |  |  |  | 0.0005 |  |  |
| Incidence × No health problems^1^ |  |  |  |  | 0.0016^*^ |  |
| Mortality × No health problems |  |  |  |  | -0.0121^*^ |  |
| Stringency × No health problems |  |  |  |  | 0.0089^*^ |  |
| Incidence × More severe health problems^2^ |  |  |  |  | 0.0001 |  |
| Mortality × More severe health problems |  |  |  |  | 0.0242^*^ |  |
| Stringency × More severe health problems |  |  |  |  | -0.0098^*^ |  |
| Incidence × Financal insecure^3^ |  |  |  |  |  | -0.0001 |
| Mortality × Financal insecure |  |  |  |  |  | 0.0030 |
| Stringency × Financal insecure |  |  |  |  |  | -0.0037^*^ |
| Observations | 64,046 | 64,046 | 64,046 | 64,046 | 64,046 | 64,046 |
| Individuals | 24,992 | 24,992 | 24,992 | 24,992 | 24,992 | 24,992 |

Note: Results from individual fixed regression, controlling for wave fixed effects. ^1^ EQ-5D sum score =5, ^2^ EQ-5D sum score >= 10, ^3^ Having some or great difficulties with making ends meet. Reference groups are age between 35 and 64, male, non-tertiary educated, some health problems (EQ-5D sum score between 6 and 9), financial secure. ^+^ *p* < 0.10, ^*^ *p* < 0.05

**Table A5** Associations of incidence, mortality and stringency with the ICECAP-A attachement dimension.

|  | Overall | Age | Gender | Education | Health | Income |
| --- | --- | --- | --- | --- | --- | --- |
| Increase in 7-day incidence by 100 per 100k | 0.0000 | 0.0001 | 0.0002 | -0.0004 | 0.0001 | 0.0002 |
| Increase in 7-day mortality by 10 per million | -0.0008 | -0.0024 | 0.0017 | -0.0027 | 0.0018 | 0.0012 |
| Increase in Stringency Index by 10 points | -0.0007 | -0.0004 | -0.0008 | -0.0003 | -0.0018 | -0.0004 |
| Incidence × Age < 35 |  | -0.0004 |  |  |  |  |
| Mortality × Age < 35 |  | 0.0075 |  |  |  |  |
| Stringency × Age < 35 |  | -0.0009 |  |  |  |  |
| Incidence × Age >= 65 |  | -0.0001 |  |  |  |  |
| Mortality × Age >= 65 |  | -0.0009 |  |  |  |  |
| Stringency × Age >= 65 |  | -0.0006 |  |  |  |  |
| Incidence × Female |  |  | -0.0002 |  |  |  |
| Mortality × Female |  |  | -0.0050 |  |  |  |
| Stringency × Female |  |  | 0.0001 |  |  |  |
| Incidence × Tertiary educated |  |  |  | 0.0007^+^ |  |  |
| Mortality × Tertiary educated |  |  |  | 0.0045 |  |  |
| Stringency × Tertiary educated |  |  |  | -0.0008 |  |  |
| Incidence × No health problems^1^ |  |  |  |  | 0.0002 |  |
| Mortality × No health problems |  |  |  |  | -0.0113^*^ |  |
| Stringency × No health problems |  |  |  |  | 0.0067^*^ |  |
| Incidence × More severe health problems^2^ |  |  |  |  | -0.0004 |  |
| Mortality × More severe health problems |  |  |  |  | 0.0105 |  |
| Stringency × More severe health problems |  |  |  |  | -0.0070^*^ |  |
| Incidence × Financal insecure^3^ |  |  |  |  |  | -0.0005 |
| Mortality × Financal insecure |  |  |  |  |  | -0.0049 |
| Stringency × Financal insecure |  |  |  |  |  | -0.0005 |
| Observations | 64,046 | 64,046 | 64,046 | 64,046 | 64,046 | 64,046 |
| Individuals | 24,992 | 24,992 | 24,992 | 24,992 | 24,992 | 24,992 |

Note: Results from individual fixed regression, controlling for wave fixed effects. ^1^ EQ-5D sum score =5, ^2^ EQ-5D sum score >= 10, ^3^ Having some or great difficulties with making ends meet. Reference groups are age between 35 and 64, male, non-tertiary educated, some health problems (EQ-5D sum score between 6 and 9), financial secure. ^+^ *p* < 0.10, ^*^ *p* < 0.05

**Table A6** Associations of incidence, mortality and stringency with the ICECAP-A autonomy dimension.

|  | Overall | Age | Gender | Education | Health | Income |
| --- | --- | --- | --- | --- | --- | --- |
| Increase in 7-day incidence by 100 per 100k | -0.0001 | 0.0001 | 0.0001 | 0.0000 | -0.0002 | 0.0003 |
| Increase in 7-day mortality by 10 per million | -0.0115^*^ | -0.0144^*^ | -0.0132^*^ | -0.0128^*^ | -0.0121^*^ | -0.0098^*^ |
| Increase in Stringency Index by 10 points | 0.0025^+^ | 0.0029^*^ | 0.0028^+^ | 0.0026^*^ | 0.0024^+^ | 0.0034^*^ |
| Incidence × Age < 35 |  | -0.0007 |  |  |  |  |
| Mortality × Age < 35 |  | 0.0064 |  |  |  |  |
| Stringency × Age < 35 |  | -0.0031^*^ |  |  |  |  |
| Incidence × Age >= 65 |  | -0.0002 |  |  |  |  |
| Mortality × Age >= 65 |  | 0.0103^+^ |  |  |  |  |
| Stringency × Age >= 65 |  | 0.0002 |  |  |  |  |
| Incidence × Female |  |  | -0.0004 |  |  |  |
| Mortality × Female |  |  | 0.0031 |  |  |  |
| Stringency × Female |  |  | -0.0006 |  |  |  |
| Incidence × Tertiary educated |  |  |  | -0.0002 |  |  |
| Mortality × Tertiary educated |  |  |  | 0.0027 |  |  |
| Stringency × Tertiary educated |  |  |  | -0.0005 |  |  |
| Incidence × No health problems^1^ |  |  |  |  | 0.0002 |  |
| Mortality × No health problems |  |  |  |  | -0.0039 |  |
| Stringency × No health problems |  |  |  |  | 0.0056^*^ |  |
| Incidence × More severe health problems^2^ |  |  |  |  | -0.0002 |  |
| Mortality × More severe health problems |  |  |  |  | 0.0155^*^ |  |
| Stringency × More severe health problems |  |  |  |  | -0.0109^*^ |  |
| Incidence × Financal insecure^3^ |  |  |  |  |  | -0.0011^*^ |
| Mortality × Financal insecure |  |  |  |  |  | -0.0041 |
| Stringency × Financal insecure |  |  |  |  |  | -0.0018^*^ |
| Observations | 64,046 | 64,046 | 64,046 | 64,046 | 64,046 | 64,046 |
| Individuals | 24,992 | 24,992 | 24,992 | 24,992 | 24,992 | 24,992 |

Note: Results from individual fixed regression, controlling for wave fixed effects. ^1^ EQ-5D sum score =5, ^2^ EQ-5D sum score >= 10, ^3^ Having some or great difficulties with making ends meet. Reference groups are age between 35 and 64, male, non-tertiary educated, some health problems (EQ-5D sum score between 6 and 9), financial secure. ^+^ *p* < 0.10, ^*^ *p* < 0.05

**Table A7** Associations of incidence, mortality and stringency with the ICECAP-A achievement dimension.

|  | Overall | Age | Gender | Education | Health | Income |
| --- | --- | --- | --- | --- | --- | --- |
| Increase in 7-day incidence by 100 per 100k | 0.0001 | 0.0004 | 0.0001 | 0.0004 | -0.0002 | 0.0002 |
| Increase in 7-day mortality by 10 per million | -0.0122^*^ | -0.0124^*^ | -0.0128^*^ | -0.0165^*^ | -0.0104^*^ | -0.0107^*^ |
| Increase in Stringency Index by 10 points | -0.0010 | -0.0010 | -0.0003 | -0.0010 | -0.0016 | -0.0003 |
| Incidence × Age < 35 |  | -0.0010^*^ |  |  |  |  |
| Mortality × Age < 35 |  | 0.0007 |  |  |  |  |
| Stringency × Age < 35 |  | 0.0005 |  |  |  |  |
| Incidence × Age >= 65 |  | -0.0005 |  |  |  |  |
| Mortality × Age >= 65 |  | -0.0010 |  |  |  |  |
| Stringency × Age >= 65 |  | -0.0001 |  |  |  |  |
| Incidence × Female |  |  | -0.0000 |  |  |  |
| Mortality × Female |  |  | 0.0012 |  |  |  |
| Stringency × Female |  |  | -0.0014 |  |  |  |
| Incidence × Tertiary educated |  |  |  | -0.0006 |  |  |
| Mortality × Tertiary educated |  |  |  | 0.0093^+^ |  |  |
| Stringency × Tertiary educated |  |  |  | -0.0005 |  |  |
| Incidence × No health problems^1^ |  |  |  |  | 0.0010^*^ |  |
| Mortality × No health problems |  |  |  |  | -0.0152^*^ |  |
| Stringency × No health problems |  |  |  |  | 0.0074^*^ |  |
| Incidence × More severe health problems^2^ |  |  |  |  | -0.0005 |  |
| Mortality × More severe health problems |  |  |  |  | 0.0239^*^ |  |
| Stringency × More severe health problems |  |  |  |  | -0.0112^*^ |  |
| Incidence × Financal insecure^3^ |  |  |  |  |  | -0.0005 |
| Mortality × Financal insecure |  |  |  |  |  | -0.0034 |
| Stringency × Financal insecure |  |  |  |  |  | -0.0016^*^ |
| Observations | 64,046 | 64,046 | 64,046 | 64,046 | 64,046 | 64,046 |
| Individuals | 24,992 | 24,992 | 24,992 | 24,992 | 24,992 | 24,992 |

Note: Results from individual fixed regression, controlling for wave fixed effects. ^1^ EQ-5D sum score =5, ^2^ EQ-5D sum score >= 10, ^3^ Having some or great difficulties with making ends meet. Reference groups are age between 35 and 64, male, non-tertiary educated, some health problems (EQ-5D sum score between 6 and 9), financial secure. ^+^ *p* < 0.10, ^*^ *p* < 0.05

**Table A8** Associations of incidence, mortality and stringency with the ICECAP-A enjoyment dimension.

|  | Overall | Age | Gender | Education | Health | Income |
| --- | --- | --- | --- | --- | --- | --- |
| Increase in 7-day incidence by 100 per 100k | -0.0004 | -0.0002 | -0.0003 | -0.0003 | -0.0006^+^ | -0.0003 |
| Increase in 7-day mortality by 10 per million | -0.0073^+^ | -0.0074^+^ | -0.0110^*^ | -0.0083^+^ | -0.0038 | -0.0053 |
| Increase in Stringency Index by 10 points | -0.0020 | -0.0021 | -0.0013 | -0.0019 | -0.0031^*^ | -0.0012 |
| Incidence × Age < 35 |  | -0.0007 |  |  |  |  |
| Mortality × Age < 35 |  | -0.0001 |  |  |  |  |
| Stringency × Age < 35 |  | -0.0001 |  |  |  |  |
| Incidence × Age >= 65 |  | -0.0001 |  |  |  |  |
| Mortality × Age >= 65 |  | 0.0007 |  |  |  |  |
| Stringency × Age >= 65 |  | 0.0004 |  |  |  |  |
| Incidence × Female |  |  | -0.0002 |  |  |  |
| Mortality × Female |  |  | 0.0073 |  |  |  |
| Stringency × Female |  |  | -0.0015 |  |  |  |
| Incidence × Tertiary educated |  |  |  | -0.0001 |  |  |
| Mortality × Tertiary educated |  |  |  | 0.0022 |  |  |
| Stringency × Tertiary educated |  |  |  | -0.0003 |  |  |
| Incidence × No health problems^1^ |  |  |  |  | 0.0009^*^ |  |
| Mortality × No health problems |  |  |  |  | -0.0146^*^ |  |
| Stringency × No health problems |  |  |  |  | 0.0080^*^ |  |
| Incidence × More severe health problems^2^ |  |  |  |  | -0.0003 |  |
| Mortality × More severe health problems |  |  |  |  | 0.0127^+^ |  |
| Stringency × More severe health problems |  |  |  |  | -0.0094^*^ |  |
| Incidence × Financal insecure^3^ |  |  |  |  |  | -0.0002 |
| Mortality × Financal insecure |  |  |  |  |  | -0.0044 |
| Stringency × Financal insecure |  |  |  |  |  | -0.0020^*^ |
| Observations | 64,046 | 64,046 | 64,046 | 64,046 | 64,046 | 64,046 |
| Individuals | 24,992 | 24,992 | 24,992 | 24,992 | 24,992 | 24,992 |

Note: Results from individual fixed regression, controlling for wave fixed effects. ^1^ EQ-5D sum score =5, ^2^ EQ-5D sum score >= 10, ^3^ Having some or great difficulties with making ends meet. Reference groups are age between 35 and 64, male, non-tertiary educated, some health problems (EQ-5D sum score between 6 and 9), financial secure. ^+^ *p* < 0.10, ^*^ *p* < 0.05
